# Inactivation of PI3K-C2α deregulates cell death pathways and sensitizes to endotoxic shock

**DOI:** 10.1073/pnas.2423358122

**Published:** 2025-07-17

**Authors:** York Posor, Sarah E. Conduit, Wayne Pearce, Daniele Morelli, Georgia Constantinou, Maria Whitehead, Neil J. Sebire, Cheryl L. Scudamore, Nieves Peltzer, Henning Walczak, Bart Vanhaesebroeck

**Affiliations:** ^a^Department of Oncology, University College London (UCL) Cancer Institute, University College London, London WC1E 6DD, United Kingdom; ^b^Molecular Pharmacology and Cell Biology, Leibniz-Forschungsinstitut für Molekulare Pharmakologie, Berlin 13125, Germany; ^c^National Institute for Health and Care Research Great Ormond Street Hospital (NIHR GOSH) Biomedical Research Centre at University College London, London WC1N 1EH, United Kingdom; ^d^Exepathology, Devon EX5 2FN, United Kingdom; ^e^Center for Molecular Medicine Cologne, University of Cologne, Cologne 50931, Germany; ^f^Excellence Cluster on Cellular Stress Responses in Aging-Associated Diseases (CECAD), University of Cologne, Cologne 50931, Germany; ^g^Department of Genome Editing, Institute of Biomedical Genetics, University of Stuttgart, Stuttgart 70569, Germany; ^h^Centre for Biochemistry, Institute of Biochemistry I, Medical Faculty, University of Cologne, Cologne 50931, Germany

**Keywords:** PI3K, regulated cell death, endotoxic shock, phosphoinositide, vascular endothelia

## Abstract

The organismal roles of the class II PI3K isoform PI3K-C2α remain poorly understood. Recent studies have found PI3K-C2α to promote arterial thrombosis and breast cancer metastasis, generating interest in this kinase as a drug target, with small molecule PI3K-C2α inhibitors now available. However, the consequences of systemic PI3K-C2α inactivation in the nondiseased, postnatal state are largely unknown. Here, we show that induction of genetic PI3K-C2α inactivation in adult mice is well tolerated, without adverse effects on normal physiology. Surprisingly, however, mice with inactive PI3K-C2α display strong sensitization to challenge with bacterial lipopolysaccharide (LPS), a model of endotoxic shock. This sensitization is recapitulated by vascular endothelial-specific deletion of PI3K-C2α. Furthermore, sensitization to LPS can be fully rescued by disabling extrinsic induction of cell death by combined caspase-8- and RIPK3 deficiency. These observations validate the tolerability of systemic PI3K-C2α inhibition in principle but reveal an unexpected role for PI3K-C2α in the regulation of extrinsic cell death pathways.

## Significance

Phosphoinositide 3-kinases (PI3Ks) phosphorylate the head group of phosphoinositide lipids in the cytoplasmic leaflet of subcellular membranes and thereby regulate signaling and membrane dynamics. The class II PI3K-C2α isoform has recently emerged as a promising drug target for antithrombotic therapies, however, the organismal roles of this PI3K in normal physiology are poorly understood. Here, we show that while inactivation of PI3K-C2α in adult mice is well tolerated, it leads to strong sensitization to the bacterial cell wall component lipopolysaccharide, a mediator of endotoxic shock during sepsis. These findings uncover a link between class II PI3Ks and the regulation of cell death pathways and are of relevance for further exploring PI3K-C2α as a drug target.

Unlike the well-characterized physiological roles of the class I and III phosphoinositide-3-kinases (PI3Ks) in metabolic signaling, nutrient sensing, angiogenesis, and immunity (1), the physiological functions of the class II PI3Ks are only beginning to emerge. The class II isoform PI3K-C2α is attracting increasing interest as a potential therapeutic target, with the first small molecule inhibitors of PI3K-C2α now having been developed (2, 3). Inactivation of PI3K-C2α has recently been shown to have potent antithrombotic effects without impairing normal hemostasis (3–5), a sought-after profile for heart attack and stroke prevention. Furthermore, overexpression of PI3K-C2α in breast cancer may promote metastasis, suggesting PI3K-C2α as a potential target for breast cancer therapy (6, 7). Despite these links to disease and inhibitors becoming available, the physiological roles of PI3K-C2α in postnatal stages as well as the consequences of systemic genetic or pharmacological inactivation of PI3K-C2α are largely unknown.

Germline loss of PI3K-C2α in mice results in embryonic lethality at mid-gestation and is associated with impaired angiogenesis (8) as well as perturbed primary cilium function (9). Mice with heterozygous inactivation due to a germline point mutation in the active site of the enzyme are viable and fertile, exhibiting mild insulin resistance in aged male mice (10) as well as impaired platelet formation and function (11). In humans, full loss of PI3K-C2α expression has been reported in a small set of patients exhibiting short stature, skeletal and neurological abnormalities, and cataract, and was accompanied by upregulation of the related class II isoform PI3K-C2β (12).

At the cellular level, PI3K-C2α has important roles in membrane remodeling and vesicular traffic (1, 13). During clathrin-mediated endocytosis, PI3K-C2α produces a pool of the phosphatidylinositol-3,4-bisphosphate [PI(3, 4)P_2_] lipid that facilitates constriction of the vesicle neck in preparation of membrane fission (14, 15). PI3K-C2α also localizes to a specific recycling endosomal compartment at the base of primary cilia to produce PI(3)P in support of ciliary function (9). Cell division has been reported to involve PI3K-C2α in two separate instances, as a scaffold protein independent of its catalytic activity in mitotic chromosome segregation (16), and in cytokinetic abscission specifically of lens epithelial cells by facilitating ESCRT-III recruitment to the midbody through formation of PI(3, 4)P_2_ (17). These observations emphasize the importance of discriminating between catalytic and scaffolding functions of PI3K-C2α.

Here, we set out to understand the physiological consequences of systemic inactivation of PI3K-C2α using mouse models that preserve the scaffolding functions of PI3K-C2α. While inactivation of PI3K-C2α in adult mice is well tolerated, we find strong sensitization to challenge with the bacterial cell wall component LPS, a model of endotoxic shock and sepsis. This effect is largely recapitulated by deletion of PI3K-C2α in vascular endothelia and depends on caspase 8- and RIPK3-mediated cell death pathways. These observations reveal an unexpected link between class II PI3Ks and cell death signaling and will need to be considered when exploring PI3K-C2α as a therapeutic target.

## Results

### Embryonic Lethality Upon PI3K-C2α Inactivation Is Associated with Features of Deregulated Cell Death Pathways.

Previous studies had associated the lethality of PI3K-C2α-deficient embryos around E10.5 to E11.5 with multiple defects during development. Indeed, Yoshioka et al. reported a defect in vascular development (8), whereas Franco et al. found slightly earlier developmental defects at E10.5 as a consequence of impaired primary cilium function and Sonic Hedgehog signaling (9).

**Fig. 1.**
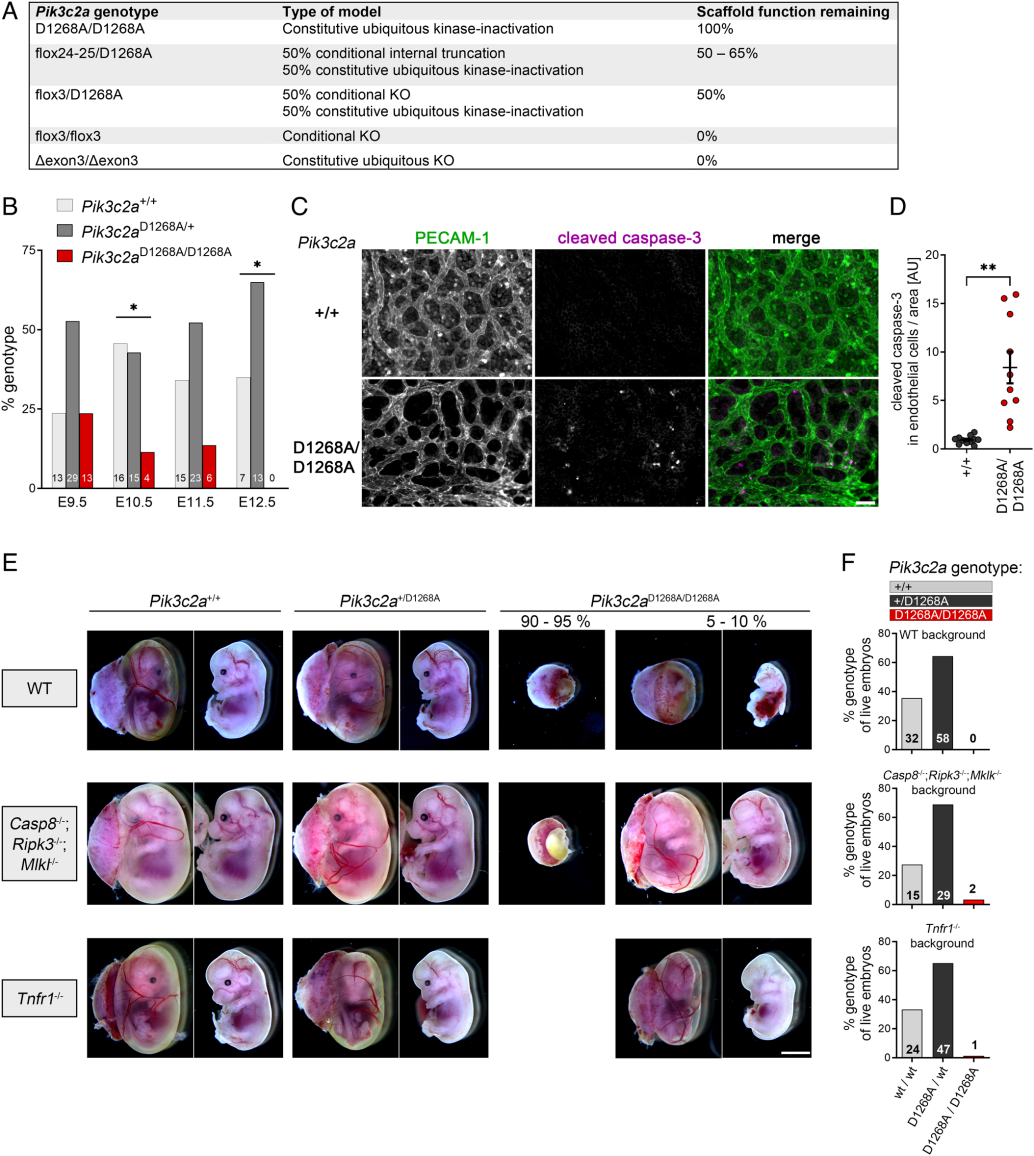
Embryonic lethal phenotype of kinase-inactive *Pik3c2a*^D1268A/D1268A^ mice shows features of deregulated cell death. Homozygous inactivation of PI3K-C2α by point mutation of Asp1268 to Ala (D1268A) is lethal at mid-gestation. (*A*) Overview of the *Pik3c2a* loss-of-function models used in this study. Note that “scaffold function remaining” refers to tissues that underwent Cre-mediated recombination and designates the amount of kinase-inactive PI3K-C2α protein expressed relative to WT PI3K-C2α protein. The internally truncated PI3K-C2α protein (Fig. 2*A*) is less stable than the WT protein. (*B*) Analysis of embryo genotypes from *Pik3c2a*^+/D1268A^ × *Pik3c2a*^+/D1268A^ matings at day of embryonic development E9.5 to E12.5. The frequency of live embryos with homozygous inactivation of PI3K-C2α is decreased at E10.5 and E11.5. At E12.5, no live *Pik3c2a*^D1268A/D1268A^ embryos were found. Figures at the *Bottom* of the bars show the number of embryos dissected and genotyped per group. Chi-square test comparing observed vs. expected genotype frequencies; *, *P* ≤ 0.05. (*C* and *D*) E10.5 yolk sacs were stained for the endothelial marker PECAM-1 and for cleaved caspase-3 as an indicator of cell death. *Pik3c2a*^D1268A/D1268A^ yolk sacs show increased cleaved caspase-3 staining in endothelial cells, as quantified in (*D*). (*C*) (Scale bar, 50 µm.) (*D*), Two-tailed t-test with Welch’s correction. **P < 0.01. (*E* and *F*) PI3K-C2α-inactive mice were crossed to *Casp8*^−/−^;*Ripk3*^−/−^;*Mlkl*^−/−^ triple KO mice (deficient for extrinsic induction of cell death) or *Tnfr1*^−/−^ mice and embryos from *Pik3c2a*^+/D1268A^ × *Pik3c2a*^+/D1268A^ matings on the respective background were analyzed at E12.5. Each embryo is shown before (*Left* panels) and after removal of yolk sac and placenta (*Right* panels). Note that most *Pik3c2a*^D1268A/D1268A^ embryos (26 out of 28 for *Casp8*^−/−^;*Ripk3*^−/−^;*Mlkl*^−/−^; 12 out of 13 for *Tnfr1*^−/−^) still showed delayed development and lethality by E12.5. However, a small minority of *Pik3c2a*^D1268A/D1268A^ embryos on the *Casp8*^−/−^;*Ripk3*^−/−^;*Mlkl*^−/−^ background and also on the *Tnfr1*^−/−^ background showed dramatically improved development and intact vascularization at E12.5. These embryos all displayed anophthalmia, a hallmark of disrupted Hedgehog signaling. (Scale bar, 3 mm.) (*F*) Numbers of dissected and genotyped embryos as in (*E*). Figures at the bottom of the bars show the number of embryos dissected and genotyped per group.

To determine the contributions of kinase activity and scaffolding functions of PI3K-C2α (16) for embryonic development, we generated homozygous *Pik3c2a*^D1268A^ mice in which PI3K-C2α is constitutively inactive due to a germline D1268A point-mutation in the *Pik3c2a* gene (10) (see Fig. 1*A* for an overview of the *Pik3c2a* loss-of-function models used in this study). Homozygous *Pik3c2a*^D1268A^ inactivation led to embryonic lethality at E10.5 to E11.5 and developmental delay (Fig. 1*B*), indistinguishable from observations made with PI3K-C2α gene knockout (KO) mice (8, 9). This indicates that during development, inactivation of kinase activity fully recapitulates loss of protein expression of PI3K-C2α.

Embryonic lethality around E11.5 accompanied by vascularization defects (8) is frequently observed in mouse strains that exhibit deregulated cell death downstream of death receptors such as tumor necrosis factor receptor-1 (TNFR-1), leading to exacerbated apoptotic and/or necroptotic cell death (18). Examples include perturbation of components of the cell death-inducing signaling complex, which can form downstream of death receptor activation, such as KO of caspase-8 (19, 20), FADD (21, 22), c-FLIP (23), or components of the linear ubiquitin chain assembly complex (LUBAC) (24, 25). The cause for embryonic lethality in these mutants is aberrant induction of apoptotic and/or necroptotic death in vascular endothelial cells. Indeed, embryonic development is restored by simultaneous disruption of both apoptotic and necroptotic pathways, such as by additional loss of RIPK3 in caspase-8 and FADD KO mice (26, 27). Similarly, embryonic lethality in mice with cFLIP or LUBAC loss-of-function can be restored by concurrent disruption of caspase-8 and RIPK3 or MLKL (24, 28, 29).

We therefore asked whether *Pik3c2a*^D1268A/D1268A^ embryos showed signs of deregulated cell death pathways. Upon inactivation of PI3K-C2α, yolk sacs of E10.5 embryos displayed a strong increase of caspase-8 activation in vascular endothelial cells, labeled by the cell-adhesion protein PECAM-1 (Fig. 1 *C* and *D*). To determine whether aberrant induction of cell death is causative of embryonic lethality, we crossed PI3K-C2α-inactive mice onto mice deficient for extrinsic induction of both apoptosis and necroptosis. Because of a potential divergent role of RIPK3 and MLKL in necroptosis-dependent and -independent functions (24, 28, 29), we decided to delete both RIPK3 and MLKL simultaneously (*Casp8*^−/−^;*Ripk3*^−/−^;*Mlkl*^−/−^). In most *Pik3c2a*^D1268A/D1268A^ embryos, ablating extrinsic cell death did not rescue embryonic development. However, a small minority of *Pik3c2a*^D1268A/D1268A^; *Casp8*^−/−^;*Ripk3*^−/−^;*Mlkl*^−/−^ embryos (2 out of 28) showed strikingly improved development with prominent vascularization of both embryo and yolk sac (Fig. 1 *E* and *F*). Such an event was never observed in *Pik3c2a*^D1268A/D1268A^ embryos on a wild-type (WT)-background (0 out of 32).

Since aberrant endothelial cell death during early embryogenesis is driven by TNFα and can be rescued by ablation of TNFR-1 (25), we also crossed PI3K-C2α-inactive mice onto *Tnfr1*^−/−^ mice. This corroborated our observations above, with only a small minority (1 out of 13) of *Pik3c2a*^D1268A/D1268A^; *Tnfr1*^−/−^ embryos showing partially rescued development (Fig. 1 *E* and *F*).

Intriguingly, all PI3K-C2α-inactive embryos that showed improved development resulting from either abrogated extrinsic cell death or TNFR-1 deficiency displayed anophthalmia (lack of one or of both eyes; Fig. 1*E*), a hallmark feature of perturbed Hedgehog signaling (30). This observation, combined with the low frequency of surviving *Pik3c2a*^D1268A/D1268A^;*Casp8*^−/−^;*Ripk3*^−/−^;*Mlkl*^−/−^ and *Pik3c2a*^D1268A/D1268A^;*Tnfr1*^−/−^ embryos, suggests that developmental delay as a consequence of disrupted Hedgehog signaling is the primary cause of lethality in *Pik3c2a*^D1268A/D1268A^ embryos, as put forward by (9). In rare cases, however, embryos may develop further regardless and encounter vascularization defects that also result in lethality. The observation that defective vascularization in *Pik3c2a*^D1268A/D1268A^ embryos can be rescued by ablating extrinsic apoptosis and necroptosis or by disrupting TNF-signaling strongly suggests that perturbed vascular development in *Pik3c2a*^D1268A/D1268A^ embryos is a consequence of deregulated endothelial cell death.

### Inactivation of PI3K-C2α in Adult Mice Is Well Tolerated.

**Fig. 2.**
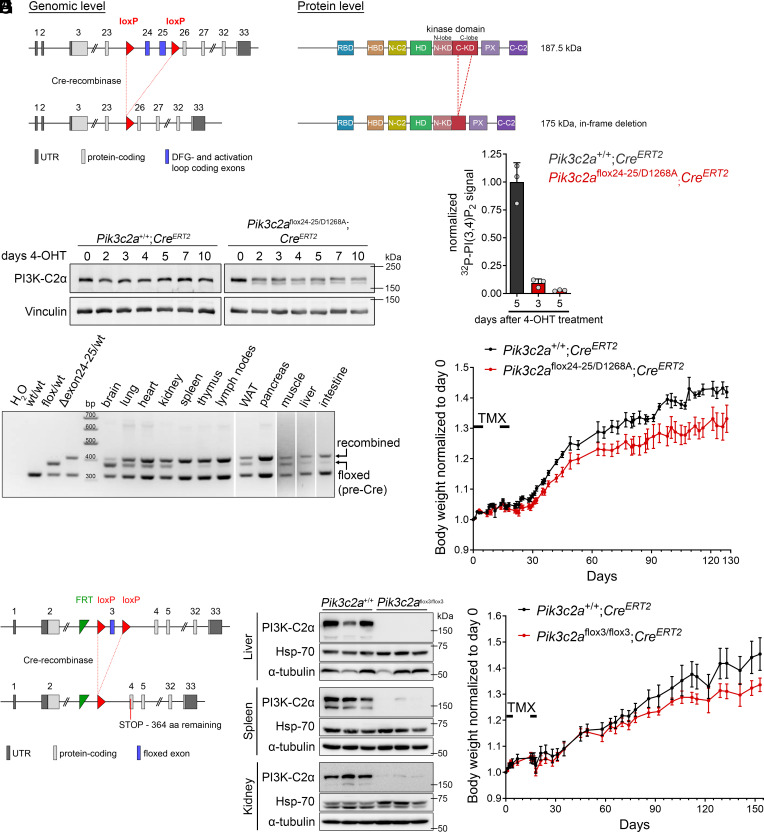
Conditional inactivation of PI3K-C2α in adult mice is well tolerated. (*A*–*E*) Conditional inactivation of PI3K-C2α in mice with one constitutively point-mutated allele (D1268A) and one allele for conditional internal truncation (flox24-25). (*A*) Scheme depicting the flox24-25 allele for conditional internal truncation of PI3K-C2α. Exons 24 and 25 of the mouse *Pik3c2a* gene are flanked by loxP sites, yielding in-frame deletion upon Cre-mediated recombination. The resulting polypeptide is expected to be 175 kDa (compared to the WT protein of 187.5 kDa) and lacks the DFG-motif and activation loop in the C-lobe of the catalytic domain. UTR, untranslated region. (*B*) Primary embryonic fibroblasts from mice with the indicated genotypes were treated with 4-OH-tamoxifen (4-OHT) for a duration of up to 10 d and protein extracts were analyzed by immunoblotting. (*C*) PI3K-C2α was immunoprecipitated from embryonic fibroblasts at 3 or 5 d after 4-OHT treatment and immunoprecipitates were assayed for kinase activity against PI(4)P using γ-^32^P-labeled ATP followed by thin layer chromatography and phosphor imager-based quantification of the amount of synthesized PI(3, 4)P_2_. Note that 5 d after 4-OHT treatment, kinase activity in *Pik3c2a*^flox24-25/D1268A^;*Cre^ERT2^* fibroblasts is nearly undetectable. (*D*) PCR-based analysis of recombination efficiency across tissues in *Pik3c2a*^flox24-25/D1268A^;*Cre^ERT2^* mice subjected to tamoxifen treatment at 8 wk of age. Cre-mediated recombination shifts the size of the PCR fragment of the floxed allele from 372 bp to 409 bp. Recombination is nearly complete in many tissues including spleen, pancreas, and lymph nodes; partial in some organs such as the lung, heart, and kidney; and largely absent in the brain. (*E*) Body weight of mice following treatment with tamoxifen (TMX) for 2 × 5 d as indicated starting at about 8 wk of age (day 0). After treatment has ended, mice resumed gaining weight. The *Pik3c2a*^flox24-25/D1268A^;*Cre^ERT2^* mice exhibited slightly reduced weight gain but showed no overt adverse phenotype. Data show body weight normalized to day 0 of treatment as mean ± s.e.m. from n = 58 (*Pik3c2a*^+/+^) or n = 46 (*Pik3c2a*^flox24-25/D1268A^) mice at the beginning of treatment. (*F*–*H*) Conditional knockout by flanking exon 3 of the mouse *Pik3c2a* gene by loxP sites (flox3). (*F*) Scheme depicting gene targeting for the flox3 allele. Deletion of exon 3 causes frame shift and premature termination in exon 4. (*G*) Liver, spleen, and kidney from mice with the indicated genotypes and treated with tamoxifen as in (*E*) were collected 6 wk after treatment onset and analyzed by immunoblotting. Hsp70 and α-tubulin shown as loading control. (*H*) Body weight of mice following treatment with tamoxifen as in (*E*). Conditional knockout of PI3K-C2α led to a tendency of reduced weight gain after treatment with tamoxifen, but no other overt adverse phenotype was observed.

To address the consequences of inactivation of PI3K-C2α at postnatal stages and to model pharmacological inhibition of PI3K-C2α in adult subjects, we created a conditional kinase-inactive mouse model for PI3K-C2α using an allele in which exons 24 and 25, encoding the ATP- and lipid substrate binding motifs, are flanked by loxP recombination sites (henceforth referred to as flox24-25). Cre recombinase-mediated excision leads to an in-frame deletion, translating to an internally truncated PI3K-C2α protein (Fig. 2*A*). To maximize efficiency and ensure the presence of at least 50% remaining intact kinase-inactive protein, we used the flox24-25 allele in combination with one point-mutated kinase-inactive D1268A allele (Fig. 1*A*). Ubiquitous conditional inactivation was achieved using a tamoxifen-inducible *Rosa26^CAG-CreERT2^* (Cre^ERT^*^2^*) line (31).

In primary mouse embryonic fibroblasts from *Pik3c2a*^flox24-25/D1268A^;*Cre^ERT2^* mice, treatment with 4-OH-tamoxifen led to appearance of a truncated form of PI3K-C2α corresponding to the gene product of the flox24-25 allele after recombination (Fig. 2*B*). This protein displayed reduced stability compared to the full-length PI3K-C2α protein, resulting in approximately 50 to 65% of total PI3K-C2α protein remaining for noncatalytic functions in *Pik3c2a*^flox24-25/D1268A^;*Cre^ERT2^* mice (Figs. 2*B* and 1*A*). Lipid kinase activity at 5 d after induction of recombination was almost completely lost (2.7 ± 0.6% of WT levels), confirming the desired outcome of the gene targeting strategy and indicating efficient recombination (Fig. 2*C*).

Inactivation of PI3K-C2α in adult mice was induced by daily administration of tamoxifen for 2× 5 consecutive days (*Materials and Methods*) and recombination assessed by PCR on genomic DNA isolated from a panel of tissues (Fig. 2*D*). Recombination efficiency varied between tissues, showing near complete recombination in many organs and less efficient recombination in the lung, heart, and kidney. The only organ tested that showed little to no recombination was the brain.

Induction of recombination did not elicit any overt adverse effects. After treatment, *Pik3c2a*^flox24-25/D1268A^;*Cre^ERT2^* mice gained weight at a reduced rate compared to *Pik3c2a*^+/+^;*Cre^ERT2^* mice (Fig. 2*E*) but exhibited no signs of stress or disease. Blood plasma from mice at 4 mo after tamoxifen treatment was subjected to clinical chemistry analysis, revealing no significant changes in any of the 22 metabolites, proteins, and ions assessed (*SI Appendix*, Fig. S1). Furthermore, histopathological examination of 25 tissues from 4 male and 4 female mice per group housed under specific pathogen-free (SPF) conditions did not show any significant morphological abnormality and there were no subjective differences in tissue morphology between WT and conditional PI3K-C2α-inactive mice (see *SI Appendix*, Table S1 for tissues included in the analysis).

To ascertain and extend these observations, we induced recombination in a cohort of mice and monitored these for the duration of 1 y, during which the mice were kept in a barrier-free holding room in open-top cages to allow for environmental pathogen exposure (*SI Appendix*, Fig. S2; pathogens present in the holding room during this period included pinworm, entamoeba, and *Pasteurella pneumotropica*). *Pik3c2a*^flox24-25/D1268A^;*Cre^ERT2^* mice showed a reduced rate of weight gain as observed under SPF conditions mentioned above without any overt adverse effects over the 12-mo period (*SI Appendix*, Fig. S2*A*). At the end of the experiment, blood plasma was analyzed for selected tissue-damage and inflammation-related parameters. This did not reveal significant alterations other than a minor decrease in albumin plasma levels, which could indicate modestly reduced liver or kidney function (32), among other potential causes (*SI Appendix*, Fig. S2*B*). To assess a possible immune impact in these mice, we analyzed myeloid (*SI Appendix*, Fig. S2*D*) and B- and T-cell populations (*SI Appendix*, Fig. S2*E*) from blood, spleen, and lymph nodes. This did not reveal any shifts in myeloid (*SI Appendix*, Fig. S2*D*) or lymphocyte (*SI Appendix*, Fig. S2*E*) subpopulations, indicating an intact immune system and the absence of acute inflammation.

Taken together, the observations above indicate that systemic inactivation of PI3K-C2α in adult mice is well tolerated. To exclude the possibility that insufficient recombination of the flox24-25 allele in some tissues (Fig. 2*D*) was obscuring potential adverse effects of PI3K-C2α inactivation in postnatal stages, we also performed classical conditional KO of PI3K-C2α, using an allele in which exon 3 of the *Pi3kc2a* gene is flanked by loxP sites, henceforth referred to as flox3 (Fig. 2*F*). To verify full loss of PI3K-C2α function after recombination of the flox3 allele, we created a germline null allele by crossing *Pik3c2a*^flox3/+^ mice onto a Cre deleter strain and intercrossed the resulting heterozygous *Pik3c2a*^Δflox3/+^ mice. These crosses did not produce any live mice with homozygous flox3-deleted (Δflox3) alleles, confirming embryonic lethality and hence full loss of function of the gene product (*SI Appendix*, Fig. S2*C*). The flox3 allele allowed us to assess efficiency of recombination at the protein level. In the liver, spleen, and kidney of *Pik3c2a*^flox3/flox3^;*Cre^ERT2^* mice, treatment with tamoxifen caused full or near complete loss of PI3K-C2α protein expression (Figs. 2G and 1A). Over the course of 5 mo after induction of recombination, these mice did not show any overt adverse phenotype and only a tendency toward reduced weight gain (Fig. 2*H*), confirming our findings using *Pik3c2a*^flox24-25/D1268A^; *Cre^ERT2^* mice.

These data establish that inactivation of PI3K-C2α in adult mice does not cause short- or long-term adverse effects and is well tolerated.

### Inactivation of PI3K-C2α Sensitizes Mice to Endotoxic Shock.

The absence of essential physiological roles of PI3K-C2α in adult mice prompted us to ask whether PI3K-C2α is required under conditions of stress. Since we observed deregulated cell death during embryonic development of *Pik3c2a*^D1268A/D1268A^ mice (Fig. 1), we turned to a model of endotoxic shock. The lethal effects of this inflammatory condition depend on cell death pathways (33–36), rendering endotoxic shock a disease-relevant model to study regulated cell death. Endotoxic shock can be elicited by the presence of bacterial lipopolysaccharide (LPS) in blood, triggering a strong and sudden innate immune response (37).

**Fig. 3.**
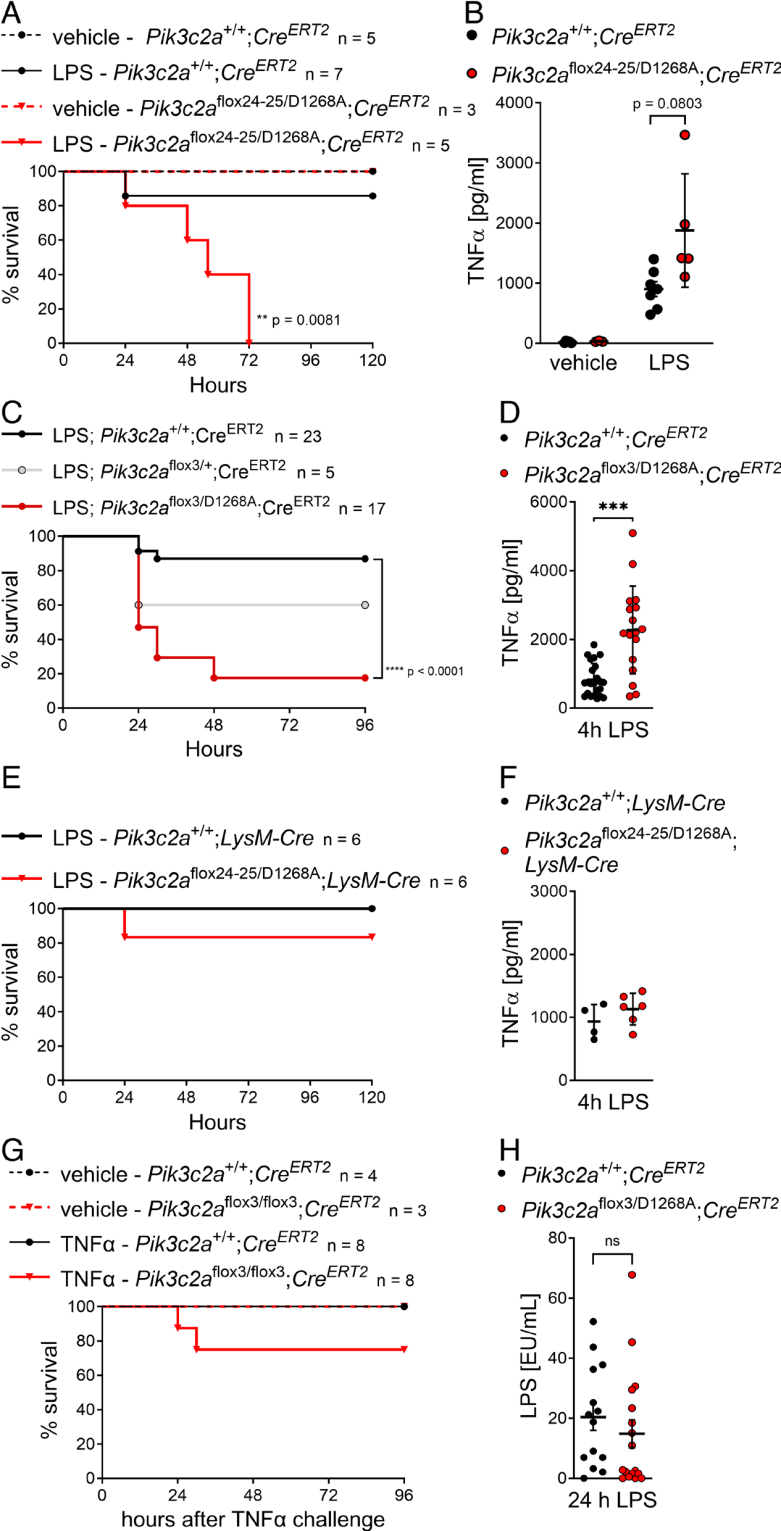
Conditional inactivation of PI3K-C2α sensitizes mice to endotoxic shock. (*A* and *B*) After global tamoxifen-induced inactivation of PI3K-C2α in adult *Pik3c2a*^flox24-25/D1268A^;*Cre^ERT2^* mice (see Fig. 2 and *Materials and Methods* for details), mice were challenged with vehicle or a sublethal dose of lipopolysaccharide (LPS; 0.5 mg/kg LPS O111:B4 by intraperitoneal injection). (*A*) Kaplan–Meier curve depicting survival of mice following LPS challenge. Inactivation of PI3K-C2α strongly sensitizes mice to LPS challenge (n denotes the number of mice per group). Data were analyzed by logrank (Mantel-Cox) test. (*B*) TNFα concentration in blood plasma obtained from mice 4 h after vehicle/LPS challenge as in (*A*), determined by bead-based 13-plex flow cytometry assay (see also *SI Appendix*, Fig. S3). Bars show mean ± s.d. and were analyzed by the two-tailed *t* test with Welch’s correction. (*C* and *D*) Global tamoxifen-induced inactivation of PI3K-C2α using the flox exon 3 allele recapitulates sensitization to LPS shown after inactivation using the flox exon 24 to 25 allele (Panel *A*). *Pik3c2a*^+/+^;*Cre^ERT2^*, *Pik3c2a*^flox3/+^;*Cre^ERT2^* or *Pik3c2a*^flox3/D1268A^;*Cre^ERT2^* mice were challenged with LPS as described for (*A* and *B*). (*C*) Survival curve following LPS challenge. Data were analyzed by logrank (Mantel-Cox) test. (*D*) TNFα in blood plasma 4 h after LPS challenge, determined by ELISA. Data are mean ± s.d. and were analyzed by the two-tailed *t* test with Welch’s correction. ***P < 0.001. (*E* and *F*) Conditional inactivation of PI3K-C2α in myeloid cells does not sensitize mice to LPS challenge. *Pik3c2a*^+/+^;*LysM-Cre* or *Pik3c2a*^flox24-25/D1268A^;*LysM-Cre* mice were challenged with LPS as described for (*A* and *B*). (*C*) Survival curve following LPS challenge. (*D*) TNFα in blood plasma 4 h after LPS challenge, determined by ELISA. Bars show mean ± s.d. (*G*) PI3K-C2α-inactive mice are not more susceptible to challenge with TNFα. After global tamoxifen-induced inactivation of PI3K-C2α in adult *Pik3c2a*^flox3/flox3^;*Cre^ERT2^* mice, mice were challenged with vehicle or a sublethal dose of TNFα (200 µg/kg murine TNFα by intravenous injection); n denotes the number of mice per group. Data were analyzed by logrank (Mantel-Cox) test, *P* = 0.2604. (*H*) LPS clearance is not affected in PI3K-C2α-inactive mice. After global tamoxifen-induced inactivation of PI3K-C2α in adult *Pik3c2a*^flox3/D1268A^;*Cre^ERT2^* mice, mice were challenged with LPS as in (*A* and *B*). Blood plasma was obtained after 24 h and LPS concentration was determined by chromogenic *Limulus* amebocyte lysate assay.

Challenge of mice with a sublethal dose of LPS to mimic endotoxic shock revealed a striking sensitization of PI3K-C2α-inactive mice to LPS. Whereas *Pik3c2a*^+/+^;*Cre^ERT2^* mice generally recovered from LPS-induced illness (weight loss, hypothermia), *Pik3c2a*^flox24-25/D1268A^;*Cre^ERT2^* mice succumbed to LPS challenge between 24 h to 72 h after injection (Fig. 3*A*). Blood plasma samples taken 4 h after injection of LPS indicated a trend toward increased levels of the major inflammatory cytokine, TNFα (Fig. 3*B*), and a trend toward increased levels of IFNβ and IL-27 in *Pik3c2a*^flox24-25/D1268A^;*Cre^ERT2^* mice (*SI Appendix*, Fig. S3). Other cytokines associated with inflammation, including the inflammasome-dependent cytokine IL-1β (38), were unchanged (*SI Appendix*, Fig. S3).

To corroborate these findings, we induced systemic inactivation of PI3K-C2α using the flox3 allele in a larger cohort of mice. We observed comparable sensitization of *Pik3c2a*^flox3/D1268A^;*Cre^ERT2^* mice to LPS challenge (Fig. 3*C*; 3 out of 23 *Pik3c2a*^+/+^;*Cre^ERT2^* mice *vs.* 14 out of 17 *Pik3c2a*^flox3/D1268A^;*Cre^ERT2^* mice succumbed to LPS challenge). Note that *Pik3c2a*^flox3/+^;*Cre^ERT2^* mice are not sensitized to the same extent as *Pik3c2a*^flox3/D1268A^;*Cre^ERT2^* mice, in agreement with a kinase-dependent function of PI3K-C2α (Fig. 3*C*). Histopathological analysis of lung, liver, and kidney sections of *Pik3c2a*^flox3/D1268A^;*Cre^ERT2^* mice that had succumbed to LPS challenge did not reveal overt tissue damage or inflammation in these tissues, with the only noticeable change being a minimal to mild increase in cellularity in the liver sinusoids of these mice (*SI Appendix*, Fig. S4 *A* and *B*). However, *Pik3c2a*^flox3/D1268A^;*Cre^ERT2^* mice had substantially increased TNFα levels, with on average 3-fold higher plasma concentrations of TNFα at 4 h after LPS challenge than the WT control group (Fig. 3*D*).

TNFα signaling is required for LPS toxicity (39) with monocytes and macrophages being the major TNFα producers upon LPS exposure (40). We therefore asked whether myeloid-lineage restricted inactivation of PI3K-C2α would recapitulate sensitization to LPS. This was not the case, given that *Pik3c2a*^flox24-25/D1268A^;*LysM-*Cre mice recovered well and did not show increased levels of TNFα upon LPS challenge (Fig. 3 *E* and *F*). This was consistent with an unchanged response to LPS stimulation in vitro of primary bone marrow–derived macrophages (as read out by TNFα secretion; *SI Appendix*, Fig. S4*C*) and dendritic cells (IL-12p40 and interferon α-secretion; *SI Appendix*, Fig. S4*D*) from *Pik3c2a*^flox24-25/D1268A^;*Cre^ERT2^* mice. These observations suggest that LPS sensitization in PI3K-C2α-inactive mice is not caused specifically by defects in TNFα-producing cells during the initiation of endotoxic shock.

We next tested whether direct challenge with recombinant TNFα (41) would recapitulate sensitization to LPS in PI3K-C2α-deficient mice. Although both groups of mice showed endotoxic shock-like symptoms such as transient hypothermia and weight loss, *Pik3c2a*^flox3/flox3^;*Cre^ERT2^* mice were not sensitized to TNFα challenge to an extent comparable to that seen for LPS, although a tendency to sensitization was observed (Fig. 3*G*). We therefore addressed the cellular response to TNFα- stimulation in immortalized embryonic fibroblasts derived from *Pik3c2a*^flox24-25/flox24-25^;*Cre^ERT2^* mice. TNF-receptor signaling appeared unaffected, with unchanged levels of pro-inflammatory NF-κB and MAPK pathway activation and no increase in the levels of cell death induction as read out by cleaved caspase-3 (*SI Appendix*, Fig. S4*E*). Since endotoxic shock is dependent on type I interferon signaling (36) and IFNβ levels showed a trend to be elevated in *Pik3c2a*^flox24-25/D1268A^;*Cre^ERT2^* mice (*SI Appendix*, Fig. S3), we also tested the response of immortalized embryonic fibroblasts to stimulation with IFNβ. Inactivation of PI3K-C2α did not change phosphorylation levels of STAT1, STAT3, and p38 (*SI Appendix*, Fig. S4*F*), indicating an unaffected transcriptional and stress signaling response to IFNβ stimulation. Whereas these data do not exclude an altered response to TNFα and IFNβ stimulation (e.g., in a tissue-specific manner), altered TNFR-1 or type I interferon receptor signaling per se are unlikely to underlie sensitization to LPS in PI3K-C2α-deficient mice.

The response to LPS challenge strongly depends on the dose. LPS clearance is a process mediated by the scavenger receptors stabilin-1/2 (42), which are internalized by clathrin-mediated endocytosis (43). In light of the importance of PI3K-C2α for clathrin-mediated endocytosis (14, 44, 45), it was conceivable that PI3K-C2α-deficient mice may experience a higher effective dose of LPS because of impaired LPS clearance. However, around the time of onset of the lethal LPS-induced effects in PI3K-C2α-deficient mice, i.e. at 24 h after LPS challenge (Fig. 3 *A* and *C*), the levels of LPS in blood plasma were not increased in *Pik3c2a*^flox3/D1268A^;*Cre^ERT2^* mice (Fig. 3*H*).

Last, we asked whether increased leakage of LPS into tissues could possibly explain our observations. Vascular permeability was assessed by Evans Blue extravasation and found to be unchanged, as measured by the amount of dye in skin and lung (*SI Appendix*, Fig. S4*G*), suggesting comparable basal vascular permeability in WT and PI3K-C2α-deficient mice.

### Loss of PI3K-C2α in Vascular Endothelia Recapitulates Sensitization to LPS.

**Fig. 4.**
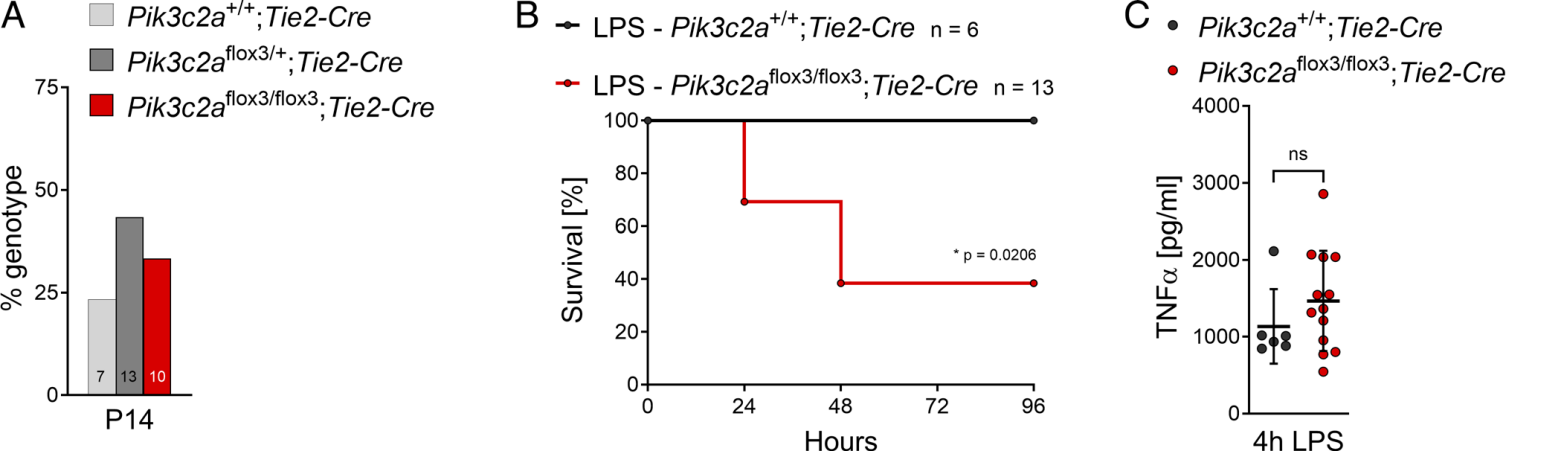
Conditional knockout of PI3K-C2α in vascular endothelial cells recapitulates sensitization to endotoxic shock. (*A*) Conditional knockout in *Pik3c2a*^flox3/flox3^ mice mediated by Cre expression under control of the vascular endothelium-specific *Tie2* promoter does not affect embryonic or perinatal survival of mice. Percentage genotype distribution at 14 d after birth (P14) from *PI3K-C2α*^flox3/+^;*Tie2-Cre* × *Pik3c2a*^flox3/+^ matings, without *Tie2-Cre*-negative offspring. The observed frequency is very close to the expected Mendelian distribution. Figures at the bottom of the bars show the number of mice genotyped per group. (*B* and *C*) *Pik3c2a*^+/+^;*Tie2-Cre* or *Pik3c2a*^flox3/flox3^;*Tie2-Cre* mice were challenged with a sublethal dose of lipopolysaccharide (LPS; 0.5 mg/kg LPS O111:B4 by intraperitoneal injection). (*B*) Kaplan–Meier curve depicting survival of mice following LPS challenge. Conditional knockout of PI3K-C2α specifically in vascular endothelial cells sensitizes mice to LPS challenge (n denotes the number of mice per group). Data were analyzed by logrank (Mantel-Cox) test. (*C*) TNFα in blood plasma 4 h after LPS challenge, determined by ELISA. Bars show mean ± s.d. and were analyzed by the two-tailed *t* test with Welch’s correction; n.s., not significant.

Loss of PI3K-C2α in myeloid cells did not recapitulate sensitization to LPS challenge (Fig. 3 *E* and *F*). Since we had observed features of deregulated cell death in endothelial cells of PI3K-C2α-deficient embryos (Fig. 1 *C* and *D*), we next focused on the vascular endothelium, a tissue previously implicated in the lethal inflammatory response to high-dose LPS challenge (35). Conditional KO of PI3K-C2α using Tie-2 promoter-directed Cre-expression to target vascular endothelia has been reported to be embryonically lethal (8). However, our experiments using the flox3 allele did not confirm this finding: When intercrossing *Pik3c2a*^flox3/wt^;*Tie2-Cre* mice, we obtained all genotypes including *Pik3c2a*^flox3/flox3^;*Tie2-Cre* mice at about the expected Mendelian frequency at P14 (Fig. 4*A*). Adult *Pik3c2a*^flox3/flox3^;*Tie2-Cre* mice did not show any overt phenotypes.

We next tested whether vascular endothelial-specific deletion of PI3K-C2α would recapitulate sensitization to LPS. By 48 h after injection, 60% of *Pik3c2a*^flox3/flox3^;*Tie2-Cre* mice but none of the WT control group had succumbed to challenge with LPS (Fig. 4*B*), and TNFα plasma levels upon LPS challenge showed a tendency to be increased (Fig. 4*C*). Hence, vascular endothelial- specific deletion of PI3K-C2α largely, if not fully, recapitulates ubiquitous inactivation of this kinase.

### Ablation of Extrinsic Cell Death Rescues Sensitization to Endotoxic Shock Upon Loss of PI3K-C2α.

**Fig. 5.**
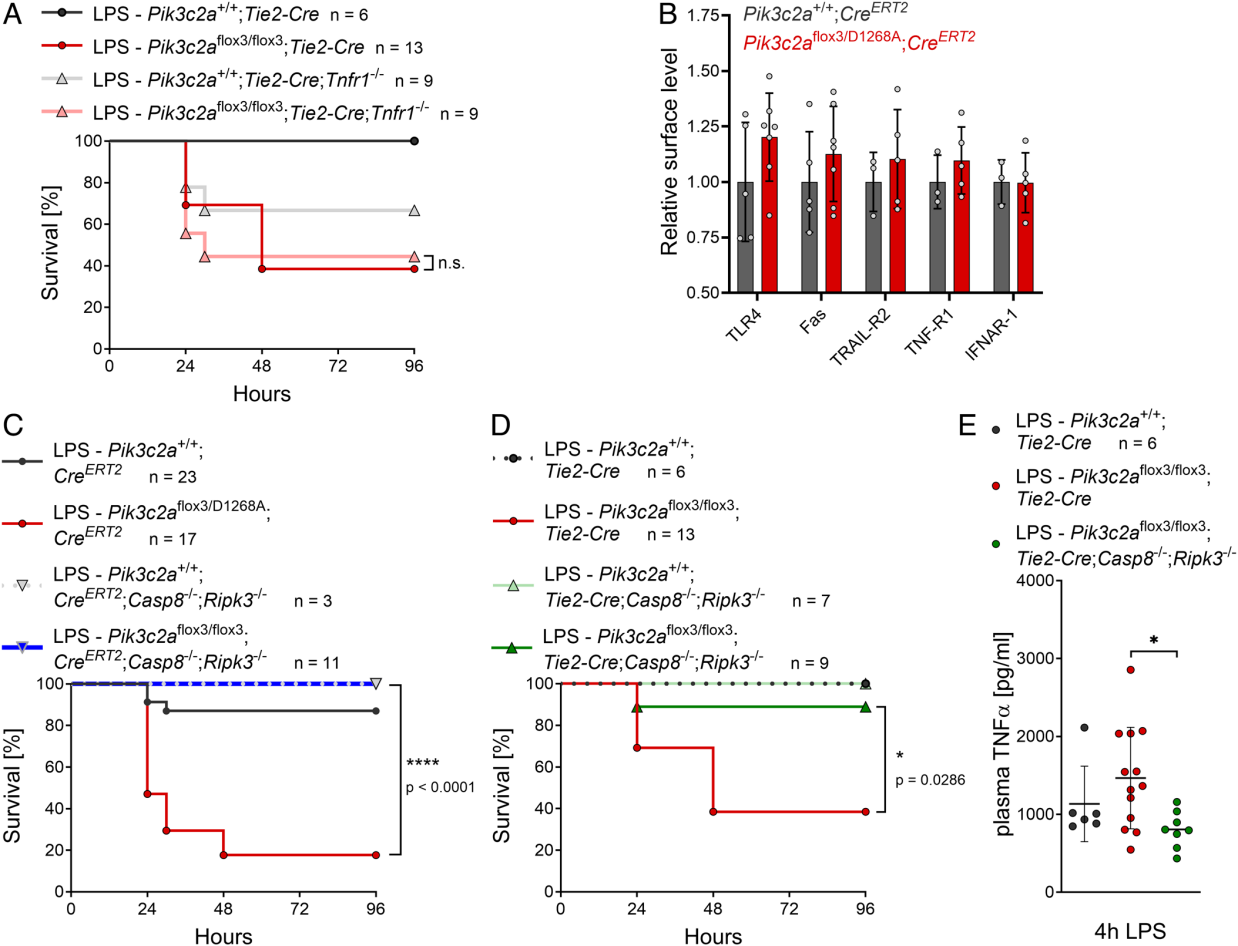
Knockout of Caspase-8 and RIPK3 rescues sensitization to endotoxic shock in PI3K-C2α knockout mice. (*A*) Knockout of TNF receptor 1 does not rescue sensitization to LPS in mice with vascular endothelial knockout of PI3K-C2α. *Pik3c2a*^+/+^;*Tie2-Cre* or *Pik3c2a*^flox3/flox3^;*Tie2-Cre* mice on a *Tnfr1*^−/−^ background were challenged with a sublethal dose of lipopolysaccharide (LPS; 0.5 mg/kg LPS O111:B4 by intraperitoneal injection). Kaplan–Meier curve depicting survival of mice following LPS challenge. Note that data for mice on TNFR-1^+/+^ background are the same as in Fig. 4*B* and shown here again for direct comparison (n denotes the number of mice per group). Data were analyzed by logrank (Mantel-Cox) test, comparing mice with PI3K-C2α knockout in vascular endothelia on *Tnfr1*^+/+^ vs. *Tnfr1*^−/−^ backgrounds. (*B*) Primary lung endothelial cells from *Pik3c2a*^+/+^;*Cre^ERT2^* or *Pik3c2a*^flox3/D1268A^;*Cre^ERT2^* mice were treated with 4-OHT to induce Cre-mediated recombination and cell surface receptor levels were measured by flow cytometry 5 to 8 d after recombination. Bars show mean fluorescence intensities ± s.d. of primary lung endothelial cell cultures obtained from n = 3 to 5 (*Pik3c2a*^+/+^;*Cre^ERT2^*) or n = 5 to 7 (*Pik3c2a*^flox24-25/D1268A^;*Cre^ERT2^*) mice. (*C*) After tamoxifen treatment at about 8 wk of age, *Pik3c2a*^+/+^;*Cre^ERT2^* or *Pik3c2a*^flox3/flox3^;*Cre^ERT2^* mice on a *Casp8*^−/−^;*Ripk3*^−/−^ background were LPS challenged as in (*A*). Kaplan–Meier curve depicting survival of mice following LPS challenge. Loss of caspase-8 and RIPK3 fully protects mice against sensitization to LPS by inactivation of PI3K-C2α. Note that data for mice on *Casp8*^+/+^;*Ripk3*^+/+^ background are the same as in Fig. 3*E* and shown here again for direct comparison (n denotes the number of mice per group). Data were analyzed by logrank (Mantel-Cox) test, comparing mice with inactive PI3K-C2α on *Casp8*^+/+^;*Ripk3*^+/+^ vs. *Casp8*^−/−^;*Ripk3*^−/−^ backgrounds. (*D* and *E*) Sensitization of mice to LPS challenge by conditional knockout of PI3K-C2α in vascular endothelia is rescued by loss of caspase-8 and RIPK3. *Pik3c2a*^+/+^;*Tie2-Cre* or *Pik3c2a*^flox3/flox3^;*Tie2-Cre* mice on a *Casp8*^−/−^;*Ripk3*^−/−^ background were challenged as in (*A*). (*D*) Kaplan–Meier curve depicting survival of mice following LPS challenge. Note that data for mice on *Casp8*^+/+^;*Ripk3*^+/+^ background are the same as in Fig. 4*B* and shown here again for direct comparison (n denotes the number of mice per group). Data were analyzed by logrank (Mantel-Cox) test, comparing mice with PI3K-C2α knockout in vascular endothelia on *Casp8*^+/+^;*Ripk3*^+/+^ vs. *Casp8*^−/−^;*Ripk3*^−/−^ backgrounds. (*E*) TNFα in blood plasma 4 h after LPS challenge, determined by ELISA. Bars show mean ± s.d. and were analyzed by ordinary one-way ANOVA and Sidak’s test; *P < 0.05.

Exacerbated cell death of endothelial cells in *Pik3c2a*^D1268A/D1268A^ mice during embryonic development appeared to be dependent on TNFR-1 (Fig. 1 *E* and *F*). To assess whether ablation of TNFR-1 would also protect PI3K-C2α-deficient endothelial cells from LPS challenge in adult mice, we crossed vascular endothelial-specific PI3K-C2α KO mice to *Tnfr1*^−/−^ mice. TNFR-1 KO did not confer any protection against low-dose LPS challenge to *Pik3c2a*^flox3/flox3^;*Tie2-Cre* mice (Fig. 5*A*). This suggests that cytokines in addition to TNFα mediate increased sensitivity to LPS in PI3K-C2α-inactive mice.

To gain further insight into the mechanistic underpinnings of LPS sensitization in endothelial cells, we isolated primary lung endothelial cells (46) from *Pik3c2a*^flox3/D1268A^;*Cre^ERT2^* mice and induced Cre-mediated recombination in vitro (*SI Appendix*, Fig. S5*A*). Based on the role of PI3K-C2α during clathrin-mediated endocytosis (14, 15, 47), we hypothesized that surface levels of cell death-inducing cytokine receptors may be increased upon PI3K-C2α inactivation, thereby possibly lowering the threshold for induction of cell death (48). PI3K-C2α-inactive lung endothelial cells did not have significantly increased surface levels of the LPS-receptor TLR4 and the death receptors Fas, TNF-R1, and TRAIL-R2, or of the surface levels of the type I interferon receptor IFNAR1 (Fig. 5*B*). Under the conditions tested, *Pik3c2a*^flox3/D1268A^;*Cre^ERT2^* primary lung endothelial cells were not sensitized to the induction of cell death in response to stimulation with LPS, alone or in combination with biologically active FasL (*SI Appendix*, Fig. S5 *B* and *C*), TNFα, or TNFα and interferon β (*SI Appendix*, Fig. S5*C*). We concluded that the diversity of endothelial cell types and the complexity of the cytokines present in blood in vivo, as well as the possible involvement of other cell types, result in the inability to accurately model LPS-sensitization in vitro at this stage. We therefore stained for cleaved caspase-3 in multiple tissues (lung, spleen, colon, liver, kidney, heart) from *Pik3c2a*^flox3/D1268A^;*Cre^ERT2^* mice after LPS exposure but did not detect cleaved caspase-3 and PECAM-1 double-positive endothelial cells (*SI Appendix*, Fig. S6). Collectively, this led us to hypothesize that LPS together with an unknown combination of cytokines is required to elicit putative exacerbated cell death in PI3K-C2α-inactive mice, possibly in a subset of endothelial cells not recapitulated by primary lung endothelial cell cultures and not identified in the tissues examined.

We therefore asked whether disabling the common mediators of cell death downstream of various death receptors, the apoptotic initiator caspase-8 and the necroptosis-inducing kinase RIPK-3 (18), would abrogate LPS sensitization in PI3K-C2α-inactive mice. Of note, caspase-8 has previously been shown to be required for high-dose LPS-induced endotoxic shock (36). Indeed, *Pik3c2a*^flox3/flox3^;*Cre^ERT2^*;*Casp8*^−/−^;*Ripk3*^−/−^ mice were fully protected from the lethal effects of LPS challenge (Fig. 5*C*). This was also the case for vascular endothelial-specific deletion of PI3K-C2α in *Pik3c2a*^flox3/flox3^;*Tie2-Cre*; *Casp8*^−/−^;*Ripk3*^−/−^ mice (Fig. 5*D*). Importantly, ablation of caspase-8 and RIPK3 also abrogated the increase in TNFα in blood plasma following LPS challenge in vascular endothelial-specific PI3K-C2α KO mice (Fig. 5*E*). Since caspase-8 and RIPK3 are not required for the initiation of endotoxic shock in hematopoietic cells (36), this suggests that the increase in TNFα may be a secondary consequence of inactivation of PI3K-C2α in endothelial cells resulting from putative exacerbated tissue damage. Taken together, these findings suggest that increased LPS sensitivity in PI3K-C2α-inactive mice may result from endothelial cells being more susceptible to cell-extrinsic triggers of apoptotic and necroptotic cell death.

## Discussion

Although the physiological roles of the class II PI3Ks are only beginning to emerge, recent findings have generated increasing interest in these enzymes as potential drug targets. Pharmacological inhibition of PI3K-C2α was shown to be antithrombotic in mice in vivo and in human blood ex vivo without impairing normal hemostasis (3), suggesting therapeutic potential in the context of heart attack and stroke prevention. Here, we describe inducible genetic inactivation of PI3K-C2α in adult mice, serving as a model of the organismal impact of pharmacological targeting of this PI3K. Ubiquitous inactivation of PI3K-C2α in adult mice up to a duration of 1 y was well tolerated without adverse effects (Fig. 2 and *SI Appendix*, Fig. S2). Whereas a modestly reduced weight gain of PI3K-C2α-inactive mice was consistent across models and experiments (Fig. 2 *E* and *H* and *SI Appendix*, Fig. S2*A*), the underlying cause remains elusive. These observations suggest that pharmacological targeting of PI3K-C2α is highly tolerable and may offer a favorable therapeutic window.

The apparent lack of essential roles of PI3K-C2α in the normal physiology of adult mice prompted us to investigate the response of PI3K-C2α-inactive mice to certain forms of stress. Since embryos with inactive PI3K-C2α show signs of deregulated endothelial cell death signaling (Fig. 1 *C*–*F*), we explored LPS-mediated endotoxic shock as a cell death-dependent model of sepsis (33–36). Indeed, ubiquitous inactivation of PI3K-C2α strongly sensitized mice to challenge with LPS (Fig. 3 *A*–*D*), a phenotype that was largely recapitulated by *Tie2*-Cre-mediated KO of PI3K-C2α in vascular endothelial cells (Fig. 4 *B* and *C*). It should be noted that *Tie2*-Cre has been reported to be also expressed in hematopoietic cells (49), yet our data do not support a contribution of the hematopoietic compartment to LPS sensitization upon PI3K-C2α inactivation: i) specific deletion of PI3K-C2α in myeloid cells using *LysM*-Cre-mediated recombination did not sensitize mice to LPS (Fig. 3 *E* and *F*), ii) bone marrow–derived macrophages and dendritic cells did not show an altered response to LPS (*SI Appendix*, Fig. S4 *C* and *D*), and iii) myeloid and lymphoid cell populations were not affected by PI3K-C2α inactivation in vivo (*SI Appendix*, Fig. S2 *D* and *E*). Although sensitization to LPS challenge was accompanied by increased levels of TNFα in blood (Fig. 3*D*), our data indicate that TNFα does not mediate this effect directly, at least not on its own (Figs. 3 *E*–*G* and 5*A*), with increased TNFα levels likely being a secondary consequence of putative exacerbated tissue damage in PI3K-C2α-inactive mice. Our data with vascular endothelial cell KO of PI3K-C2α points to an endothelial cell-intrinsic mechanism to underlie sensitization to LPS. In line with this interpretation, we found that disabling the pathways that trigger extrinsic cell death in response to death receptor activation (18) by double KO of caspase-8 and RIPK3 fully rescued sensitization to LPS in PI3K-C2α-deficient mice (Fig. 5 *C* and *D*). These findings support a model whereby inactivation of PI3K-C2α predisposes endothelial cells to undergo apoptosis upon stimulation with cell death-inducing cytokines induced by LPS.

It is important to note that we have not been able to directly observe increased cell death of endothelial cells upon inactivation of PI3K-C2α (*SI Appendix*, Figs. S5*C* and S6) and that both RIPK3 and caspase-8 have been reported to have cell-death independent functions in the control of inflammatory cytokine secretion (50–52). However, caspase-8 and RIPK3 are not required for the LPS-mediated initiation of endotoxic shock by the hematopoietic compartment, even though they do contribute to amplification of the inflammatory response (36). This argues for an essential role of caspase-8 and RIPK3 in LPS sensitization in PI3K-C2α-inactive mice by mediating cell death during later stages of endotoxic shock, likely in a specific vascular endothelial compartment. It needs to be taken into consideration that most published studies on LPS-mediated endotoxic shock used a high dose of LPS of 50 mg/kg or more, which conceivably may trigger widespread tissue damage (34–36, 53). We, however, have observed sensitization to LPS at doses as low as 0.5 mg/kg. This means that it may be incomparably more difficult to detect alterations at the histopathological level. Taken together, our observations are most compatible with a model whereby a yet elusive specific subset of endothelial cells is responsible for LPS sensitization. Still, we cannot strictly rule out cell death-independent contributions of caspase-8 and RIPK3 and have not found direct evidence of endothelial cell death upon LPS challenge.

Endotoxic shock requires the concerted activity of multiple cytokines, most notably TNFα and IFNβ (36), yet the full complement of signals contributing to the lethal effects of systemic LPS challenge is unknown. For example, Fas (CD95) and Fas ligand (FasL) expression are upregulated in response to LPS (54, 55) and can drive disseminated endothelial cell death (56). We tested several combinations of cytokines for their ability to induce cell death in primary lung endothelial cells in vitro, including TNFα, IFNβ, and FasL, but were unable to recapitulate sensitization to LPS challenge as observed in vivo (*SI Appendix*, Fig. S5). This could be explained by more complex combinations of cytokines, specific temporal dynamics of cytokine secretion or additional cell types that may be required to elicit endothelial cell death in vivo. Additionally, only specific subsets of endothelial cells from tissues other than lung may be more susceptible to LPS-induced cell death upon inactivation of PI3K-C2α.

What is the mechanistic basis for the increased sensitivity of PI3K-C2α-deficient vascular endothelia to LPS? A peculiar and to our knowledge unexplained observation is that vascular endothelia appear to be particularly sensitive to perturbations of death receptor signaling (24, 25, 28), as also indicated by our observations (Figs. 1 *C* and *D* and 4). This may be related to the direct exposure to cytokines circulating in the blood, but could also have more intricate underlying causes related to tissue-specific death receptor regulation by, for example, membrane trafficking. Interestingly, disruption of PI3K-C2α function has a strong bias for affecting endothelial cells. Internalization and consequently signaling of multiple receptors, including those for vascular endothelial growth factor (8), sphingosine-1-phosphate (57), and TGFβ (44, 58), has been shown to be impaired in endothelial cells upon PI3K-C2α loss. This raises the possibility that the potentially increased sensitivity of PI3K-C2α-deficient vascular endothelia to LPS-induced extrinsic apoptosis (Fig. 5*D*) is caused by impaired clathrin-mediated endocytosis (14), resulting in increased cell surface levels of death receptors and increased sensitivity to extrinsic apoptosis (48). However, using primary lung endothelial cells as a model, we have not observed significantly altered surface levels of cell death-inducing receptors (Fig. 5*B*). Further studies will have to determine the subset of endothelial cells that is primarily affected with regard to LPS sensitivity and whether deregulation of death receptor surface levels may underlie the observed sensitivity of PI3K-C2α-inactive mice to challenge with LPS.

The deleterious effects of systemic high-dose LPS exposure are in part mediated by the cytoplasmic LPS sensors, caspase-4/5 in humans, and caspase-11 in mice, leading to activation of the pore-forming protein gasdermin D, pyroptotic cell death, and release of IL-1β (33–35, 53). Several observations argue against an involvement of pyroptotic cell death in increased LPS-sensitivity upon inactivation of PI3K-C2α. Pyroptosis is typically associated with secretion of the pro-inflammatory cytokine IL-1β, which we did not observe in LPS-challenged PI3K-C2α-inactive mice (*SI Appendix*, Fig. S3). The strict dependence of LPS-sensitization on caspase-8 and RIPK3 (Fig. 5 *C* and *D*) further suggests that endothelial cell death is more likely to occur by extrinsic apoptosis. Last, the LPS doses typically employed in studies addressing cytoplasmic LPS sensors (33, 35, 53) are ~100-fold higher than those used in this study to assess sensitization to LPS (0.5 mg/kg LPS), possibly rendering penetration into the cytoplasm less likely (59).

While systemic inactivation of PI3K-C2α induced in adult mice is well tolerated, sensitization to endotoxic shock may represent a concern when considering therapeutic targeting of PI3K-C2α, as immune reactions to septic foci under conditions of PI3K-C2α inhibition would be expected to be more severe. It should be noted that a bolus injection of LPS in mice has several shortcomings as a model for sepsis in humans. While humans are intrinsically far more sensitive to LPS than mice, a bolus injection of LPS triggers a much sharper and more intense spike of pro-inflammatory cytokines such as TNFα as compared to a septic focus (60). This may explain why therapeutic interventions validated in mouse models of sepsis have often proven ineffective for treating human sepsis (61), yet it does not allow a clear prediction how sensitization to LPS as observed in PI3K-C2α-inactive mice would translate to humans. Studies using LPS-mediated endotoxic shock have fundamentally contributed to our understanding of the mechanistic and genetic basis of fatal sepsis and have allowed to identify central mediators of the lethal effects of endotoxic shock, such as caspase-11 (33, 34) and caspase-8 (36). These key findings have been confirmed in the translationally more relevant CLP model (35, 62, 63). Our findings may therefore have important implications for the development of PI3K-C2α inhibitors and need to be taken into consideration when exploring potential therapeutic targeting of this PI3K isoform.

## Materials and Methods

Please see *SI Appendix*, *Extended methods*.

### Antibodies.

Primary antibodies used in this study are listed in *SI Appendix*, Table S2. Secondary HRP-coupled antibodies against mouse or rabbit IgG for immunoblotting (GE Healthcare) were used at 1:2000 to 1:5000 dilution. Secondary antibodies for immunohistochemistry were coupled to AlexaFluor488, −568, or −647 dyes (ThermoFisher Scientific) and were used at 1:400 dilution.

### Mice.

All mice were maintained at University College London in accordance with The Animals (Scientific Procedures) Act 1986 Amendment Regulations 2012 (approved by the Animal Welfare and Ethical Review Body (AWERB), P434BB714, and PP5281579). Mice were maintained in specific pathogen-free conditions and individually ventilated cages with a 07:30 to 19:30 light and 19:30 to 07:30 dark cycle at 18 to 22 °C and 40 to 60% humidity. A single cohort of mice was kept in a conventional facility for 1 y after induction of recombination.

*Pik3c2a*^D1268A^ mice carrying the *Pik3c2a*^tm1521(D1268A)Arte^ allele have been described previously (10). Mouse gene targeting for *Pik3c2a*^flox24-25^ mice carrying the C57BL/6J-Pik3c2a^tm2Bvan^/H allele was performed by Taconic Biosciences (Cologne, Germany), using homologous recombination-based genomic editing of C57BL/6N embryonic stem cells. The targeting vector was designed to insert a puromycin selection cassette flanked by FRT sites in intron 23, which was later removed by Flp-mediated recombination, as well as loxP sites flanking exons 24 and 25 for in-frame deletion of these exons.

*Pik3c2a*^flox3^ mice carrying the *Pik3c2a*^tm1c(EUCOMM)Hmgu^ allele were obtained through the international mouse phenotyping consortium via The Centre for Phenogenomics (Toronto, ON, Canada). Note that the nomenclature of the mouse *Pik3c2a* gene exons has been inconsistent in the published literature. The first exon of the *Pik3c2a* gene is not translated, with the ATG start codon present in exon 2. The exon flanked by loxP sites in the *Pik3c2a*^tm1c(EUCOMM)Hmgu^ allele is the exon following the exon containing the ATG translation start site, i.e., in total the third exon.

The *Rosa26^CAG-CreERT2^* mouse strain expresses the Cre^ERT2^ protein under control of the CAG-promoter from the *Rosa26* locus (31). *LysM-Cre* mice carrying the Lyz2^tm1(cre)Ifo^ allele were obtained from Michael Karin (UC San Diego, CA). *Casp8*^−/−^ mice carrying the Casp8^tm1.1Raz^ allele (64), *Ripk3*^−/−^ mice carrying the Ripk3^tm1Vmd^ allele (65), and *Mlkl*^−/−^ mice (24) have been described previously. *Tnfr1*^−/−^ (strain #002818, B6.129-Tnfrsf1a^tm1Mak^/J) and *Tie2-Cre* mice (strain #008863, B6.Cg-Tg(Tek-cre)1Ywa/J) were obtained through JAX.

All primers for mouse genotyping by PCR are listed in *SI Appendix*, Table S3.

### LPS Endotoxic Shock Model.

LPS O111:B4 from *E. coli* (Sigma-Aldrich, L3012) was dissolved in sterile 0.9% NaCl solution and diluted to a working concentration of 62.5 µg/mL in sterile PBS. The experimenter was blinded to the genotype of the mice. In case of mice having previously been treated with tamoxifen, they were given at least 10 d of recovery between the last tamoxifen dose and the LPS challenge. Mice were challenged with 0.5 mg/kg LPS by intraperitoneal injection. 4 h after injection, 80 to 100 µL of blood were sampled from the tail vein using heparin-coated glass capillaries (Hirschmann Laborgeräte, Germany). Blood plasma was prepared by centrifuging for 5 min at 5,100×*g* at 4 °C and collecting the supernatant. Mice were monitored closely over a period of 96 h or 120 h and their body weight was recorded twice daily. Mice were killed if they were about to lose more than 20% of their body weight as compared to before the injection or if they appeared clearly moribund, i.e., exhibiting severe hypothermia, hunching, and being unresponsive to touch.

### Cytokine Measurement in Blood Plasma.

Cytokine levels in blood plasma were measured by ELISA or bead-based flow cytometry assay. Murine TNFα was measured using the OptEIA mouse TNF ELISA set II (BD, 558534) according to the manufacturer’s instructions. Murine IL-12p40 was measured using the OptEIA Mouse IL-12 p40 ELISA set (BD, 555165) according to the manufacturer’s instructions. Murine IFNα was measured using the Verikine mouse IFNα ELISA kit (Verikine, 42120-1) according to the manufacturer’s instructions.

For multiplexed determination of cytokines from mouse blood plasma, the LegendPlex Mouse Inflammation Panel (BioLegend, 740446) was used according to the manufacturer’s instructions and samples were measured on a BD Fortessa flow cytometer.

### Determination of LPS Concentration in Blood Plasma.

LPS concentrations were measured in mouse blood plasma using the LAL chromogenic endpoint assay (HycultBiotech, HIT302) according to the manufacturer’s instructions, including centrifugation of the collected blood to separate plasma within 20 min of sampling.

### Clinical Chemistry Analysis of Blood Plasma.

For clinical chemistry analysis of mouse blood plasma, total blood was collected from mice under terminal anesthesia by cardiac puncture, using 25 µL of lithium-heparin (1,000 IU/mL; Sigma-Aldrich, H0878-100KU) as anticoagulant per mouse. Blood samples were centrifuged at 400×*g* for 5 min to remove cells and the supernatant was collected as blood plasma. Clinical chemistry analysis was performed at the Clinical Pathology Laboratory of the Mary Lyon Centre at MRC Harwell, UK.

### Histopathological Analysis of Tissue Sections.

Mouse tissues were harvested after the animals had been killed and fixed in 10% formalin for 24 h and then processed and embedded into paraffin wax blocks. The organs were orientated with the largest surface down, to obtain complete sections, and 3-µm serial sections were cut and stained using hematoxylin and eosin. Each section was subjectively evaluated by an experienced diagnostic histopathologist using routine light microscopy to screen for major tissue pathologies such as inflammation or neoplasia affecting any organ. Histopathological examination was performed entirely blinded to group.

### Evans Blue Vascular Permeability Assay.

Basal vascular permeability was assayed by Evans Blue leakage into tissues. A solution of 10 mg/mL Evans Blue (Sigma-Aldrich, E2129) in PBS was sterile-filtered and injected at 40 mg/kg Evans Blue into the mouse tail vein. 30 min after injection, mice were killed and tissue samples collected and weighed. Tissues were incubated in 200 µL formamide per 100 mg tissue for 24 h at 55 °C. Debris was pelleted by centrifuging at 20,000×*g* for 5 min and supernatants were collected. The amount of Evans Blue per mg tissue was determined using a standard curve and measuring the absorbance at 620 nm.

### Statistics.

Statistical analyses were performed with Graphpad Prism 10 software and details of all statistical analyses are provided in the respective figure legends. For all tests, the levels of statistical significance were defined as *P < 0.05, **P < 0.01, ***P < 0.001, and ****P < 0.0001. When two experimental conditions or groups were being compared, we used an unpaired two-tailed Student’s *t* test with Welch’s correction to compensate for the possibility of unequal variances in the two conditions. For comparisons between more than two experimental conditions, we used ANOVA followed by Šídák’s test (for multiple pairwise comparisons) to compute *P* values. *P* values from ANOVAs are reported as multiplicity-adjusted *P* values. Kaplan–Meier survival curves were analyzed by logrank (Mantel-Cox) test. Discrepancies between observed and expected values in the distribution of genotypes were analyzed by the chi-square test.

## Supplementary Material

Appendix 01 (PDF)

## Data Availability

All study data are included in the article and/or *SI Appendix*.
