# Supplementary material for: Inactivation of PI3K-C2α deregulates cell death pathways and sensitizes to endotoxic shock
Source: Proc Natl Acad Sci U S A. 2025 Jul 17;122(29):e2423358122. doi: 10.1073/pnas.2423358122 (PMC12304892; doi:10.1073/pnas.2423358122)
Supplement: Supplementary file 1 — Appendix 01 (PDF) [file pnas.2423358122.sapp.pdf]

PNAS

Supporting Information for

Inactivation of PI3K-C2α deregulates cell death pathways and sensitizes to endotoxic shock

York Posor, Sarah E. Conduit, Wayne Pearce, Daniele Morelli, Georgia Constantinou, Maria Whitehead, Neil J. Sebire, Cheryl L. Scudamore, Nieves Peltzer, Henning Walczak, Bart Vanhaesebroeck

Correspondence: York Posor, Bart Vanhaesebroeck
Email: posor@fmp-berlin.de or bart.vanh@ucl.ac.uk

This PDF file includes:

Figures S1 to S6
Tables S1 to S4
Extended methods

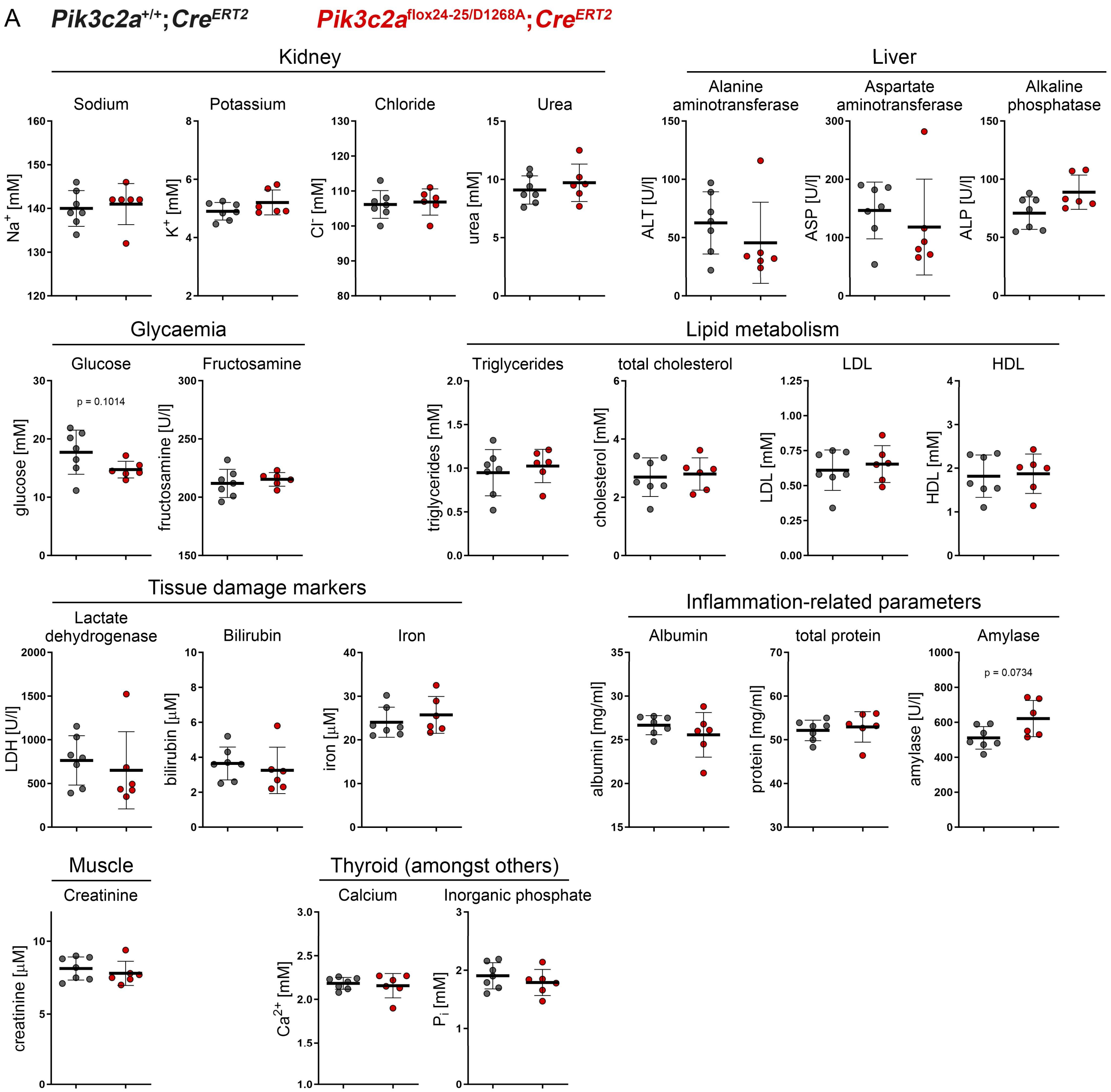

Fig. S1: Clinical chemistry analysis of blood plasma at four months after conditional inactivation of PI3K-C2α.

Lithium-heparinized blood was collected by cardiac puncture from mice at 4 months after completion of tamoxifen treatment as in Fig. 2D,E (onset of treatment in mice of about 8 weeks of age) and plasma was subjected to clinical chemistry analysis. Analytes are tentatively grouped according to the organ / process they are primarily used to report on but can be relevant in other physiological contexts. LDL, low density lipoprotein; HDL, high density lipoprotein. Data shown are mean ± s.d. from n = 7 (*Pik3c2a*$^{+/+}$;*Cre*^{ERT2}) or n = 6 (*Pik3c2a*$^{flox24\text{-}25/D1268A}$;*Cre*^{ERT2}) mice. Data for glucose and amylase were analyzed by Mann-Whitney test and the p-value is reported in the figure.

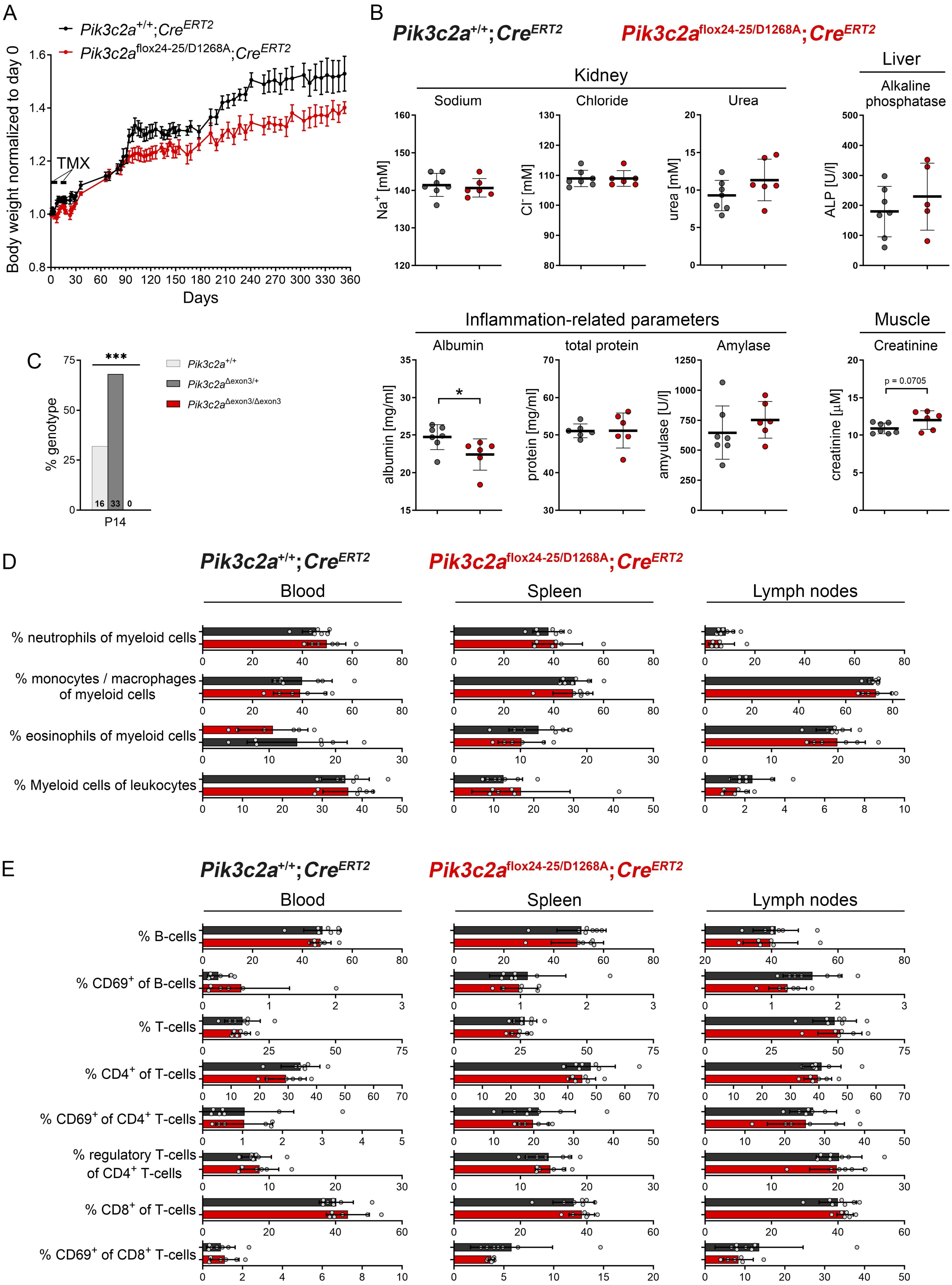
A
Pik3c2a+/+;CreERT2
Pik3c2aflox24-25/D1268A;CreERT2
Body weight normalized to day 0
TMX
Days
B
Kidney
Sodium
Chloride
Urea
Liver
Alkaline phosphatase
Inflammation-related parameters
Albumin
total protein
Amylase
Muscle
Creatinine
p = 0.0705
C
% genotype
P14
Pik3c2a+/+
Pik3c2aΔexon3/+
Pik3c2aΔexon3/Δexon3
D
Blood
Spleen
Lymph nodes
% neutrophils of myeloid cells
% monocytes / macrophages of myeloid cells
% eosinophils of myeloid cells
% Myeloid cells of leukocytes
E
% B-cells
% CD69+ of B-cells
% T-cells
% CD4+ of T-cells
% CD69+ of CD4+ T-cells
% regulatory T-cells of CD4+ T-cells
% CD8+ of T-cells
% CD69+ of CD8+ T-cells

Fig. S2. Analysis of mice aged for one year upon conditional inactivation of PI3K-C2α. Mice at the age of 8 weeks were treated with tamoxifen and monitored for about one year after onset of treatment. (A) Body weight of mice following treatment with tamoxifen (TMX) for 2 × 5 days as indicated starting at about 8 weeks of age (day 0). After treatment has ended, mice resumed gaining weight. The *Pik3c2a*$^{flox24\text{-}25/D1268A}$;*Cre*^{ERT2} mice exhibited slightly reduced weight gain but showed no overt adverse phenotype until one year after recombination. Data show body weight normalized to day 0 of treatment as mean ± s.e.m. from n = 10 (*Pik3c2a*$^{+/+}$) or n = 6 (*Pik3c2a*$^{flox24\text{-}25/D1268A}$) mice at the beginning of treatment. (B) Clinical chemistry analysis of blood plasma collected from mice about one year after onset of tamoxifen treatment as in (A). Analytes are tentatively grouped according to the organ / process they are primarily used to report on but can be relevant in other physiological contexts. Data are mean ± s.d. from n = 7 (*Pik3c2a*$^{+/+}$;*Cre*^{ERT2}) or n = 6 (*Pik3c2a*$^{flox24\text{-}25/D1268A}$;*Cre*^{ERT2}) mice. Data for albumin and creatinine were analyzed by Mann-Whitney test. *, $p < 0.05$. (C) Analysis of obtained genotypes from *Pik3c2a*$^{\Delta flox3/+}$ × *Pik3c2a*$^{\Delta flox3/+}$ matings at post-natal day 14. No homozygous *Pik3c2a*$^{\Delta flox3/\Delta flox3}$ embryos were found. Figures at the bottom of the bars show the number of mice genotyped per group. Chi-square test comparing observed vs. expected genotype frequencies, two-tailed p-value = 0.0003. (D, E) Flow cytometric analysis of myeloid cell populations (D) and B- and T-cell populations (E) from blood, spleen and lymph nodes of mice about one year after onset of tamoxifen treatment as in (A) does not reveal overt alterations upon inactivation of PI3K-C2α. Data are mean ± s.d. from n = 7 (*Pik3c2a*$^{+/+}$;*Cre*^{ERT2}) or n = 6 (*Pik3c2a*$^{flox24\text{-}25/D1268A}$;*Cre*^{ERT2}) mice. CD69 was used as a marker of activated lymphocytes. Regulatory T-cells are $CD4^{+}CD25^{+}FoxP3^{+}$ T-cells.

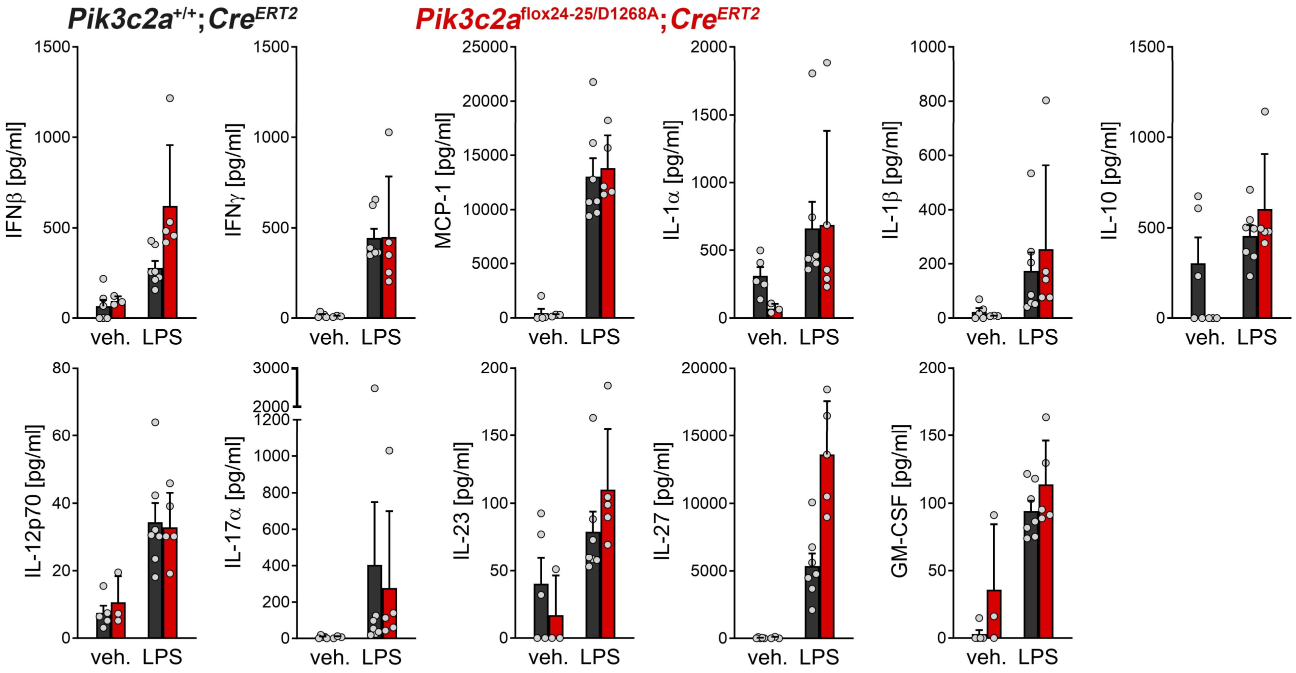

Fig. S3. Cytokine profiling in mice challenged with LPS. Cytokine concentrations in blood plasma obtained from mice 4 h after vehicle / LPS challenge as in Fig. 3A, determined by bead-based 13-plex flow cytometry assay. Bars show mean + s.d..

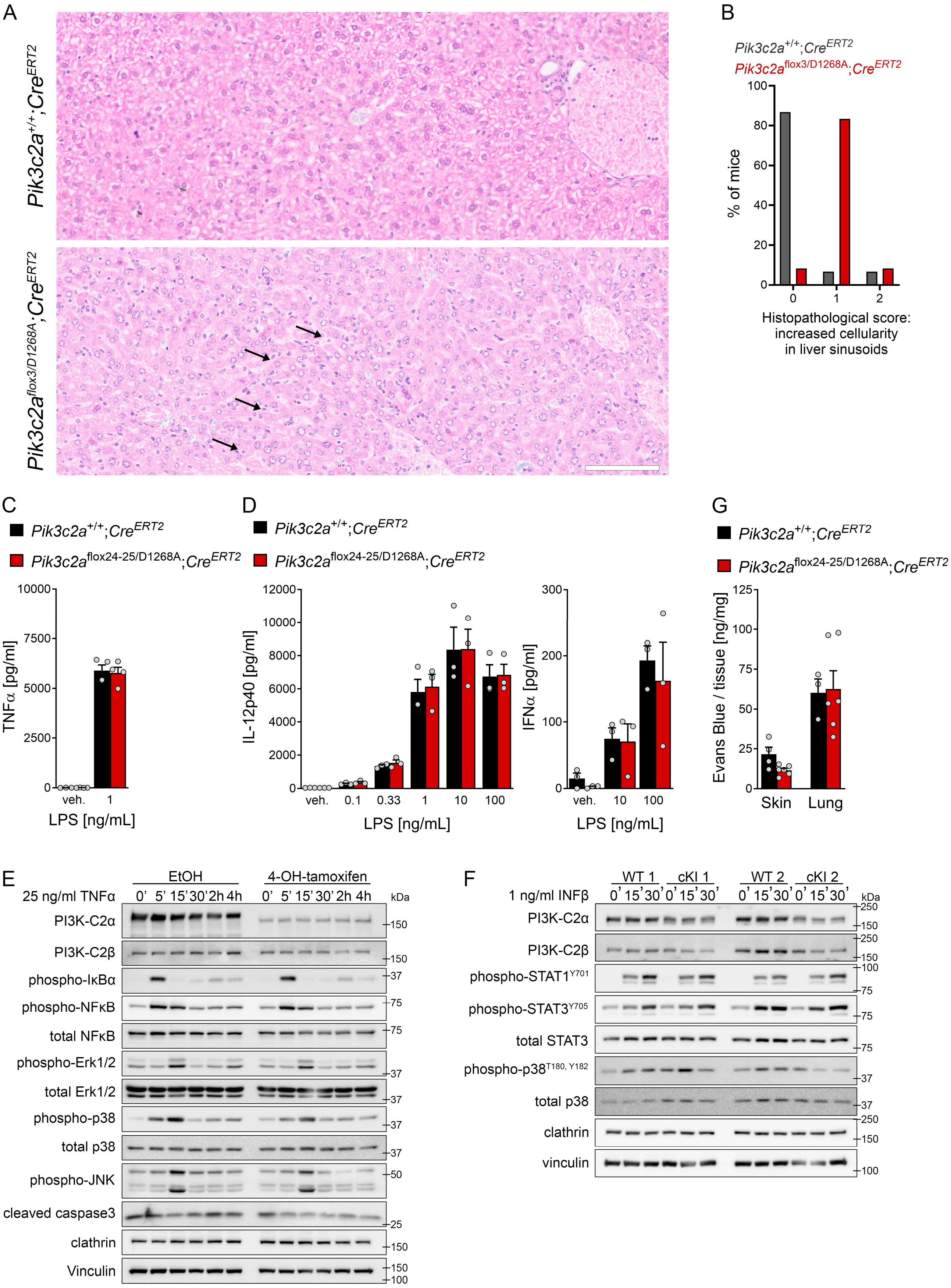
A
Pik3c2a+/+;CreERT2
Pik3c2aflox3/D1268A;CreERT2
B
Pik3c2a+/+;CreERT2
Pik3c2aflox3/D1268A;CreERT2
% of mice
Histopathological score: increased cellularity in liver sinusoids
C
Pik3c2a+/+;CreERT2
Pik3c2aflox24-25/D1268A;CreERT2
TNFα [pg/ml]
veh.
LPS [ng/mL]
D
Pik3c2a+/+;CreERT2
Pik3c2aflox24-25/D1268A;CreERT2
IL-12p40 [pg/ml]
IFNα [pg/ml]
LPS [ng/mL]
G
Pik3c2a+/+;CreERT2
Pik3c2aflox24-25/D1268A;CreERT2
Evans Blue / tissue [ng/mg]
Skin
Lung
E
25 ng/ml TNFα
EtOH
4-OH-tamoxifen
0' 5' 15' 30' 2h 4h
kDa
PI3K-C2α
PI3K-C2β
phospho-IκBα
phospho-NFκB
total NFκB
phospho-Erk1/2
total Erk1/2
phospho-p38
total p38
phospho-JNK
cleaved caspase3
clathrin
Vinculin
F
1 ng/ml INFβ
WT 1
cKI 1
WT 2
cKI 2
0' 15' 30'
kDa
PI3K-C2α
PI3K-C2β
phospho-STAT1Y701
phospho-STAT3Y705
total STAT3
phospho-p38T180, Y182
total p38
clathrin
vinculin

Fig. S4. The response to LPS, TNFα and IFNβ stimulation upon conditional inactivation of PI3K-C2α. (A, B) Histopathological analysis of mice upon LPS challenge. *Pik3c2a*$^{wt/wt}$;*Cre*^{ERT2} (n = 15) and *Pik3c2a*$^{flox3/D1268A}$;*Cre*^{ERT2} (n = 12) mice were challenged with LPS. Mice were sacrificed when they became moribund or 96 h after injection if LPS challenge was tolerated. Tissues were stained with hematoxylin / eosin. (A) Representative images from liver sections. Arrows point to mildly increased cellularity in liver sinusoids. Bar, 100 µm. (B) Histopathological scoring of cellularity in liver sinusoids in stained liver sections. Histopathological scores: 0 – normal; 1 – mild; 2 – moderate. (C, D) Cytokine secretion in response to LPS stimulation is unchanged in bone marrow-derived macrophages (C, TNFα secretion) and bone marrow-derived dendritic cells (D, IL-12p40 and IFNα secretion). Data are mean + s.e.m. from primary cultures derived from n = 4 mice per group (C) and n = 3 mice per group (D). (E) Signaling response to TNFα stimulation in *Pik3c2a*$^{flox24\text{-}25/flox24\text{-}25}$;*Cre*^{ERT2} immortalized mouse embryonic fibroblasts. Cultures were treated with vehicle (EtOH) or 4-OH-tamoxifen to induce Cre-mediated recombination and stimulated with 25 ng/mL murine TNFα for the indicated time. Cell extracts were analyzed by immunoblotting. Clathrin and vinculin were detected as loading controls. (F) Signaling response to IFNβ stimulation of *Pik3c2a*$^{wt/wt}$;*Cre*^{ERT2} (WT 1, WT2) and *Pik3c2a*$^{flox24\text{-}25/D1268A}$;*Cre*^{ERT2} (cKI 1, cKI 2) immortalized mouse embryonic fibroblast from two mice per genotype. Cultures were treated with 4-OH-tamoxifen to induce Cre-mediated recombination and stimulated with 1 ng/mL murine IFNβ for the indicated time. Cell extracts were analyzed by immunoblotting. Clathrin and vinculin were detected as loading controls. (G) Vascular permeability was assessed by tail vein injection of Evans Blue dye and determination of dye levels in skin and lung tissue (Miles assay). Global tamoxifen-induced inactivation of PI3K-C2α in adult *Pik3c2a*$^{flox24\text{-}25/D1268A}$;*Cre*^{ERT2} mice does not increase basal vascular permeability. Bars show mean + s.e.m. from n = 3 - 6 mice per group.

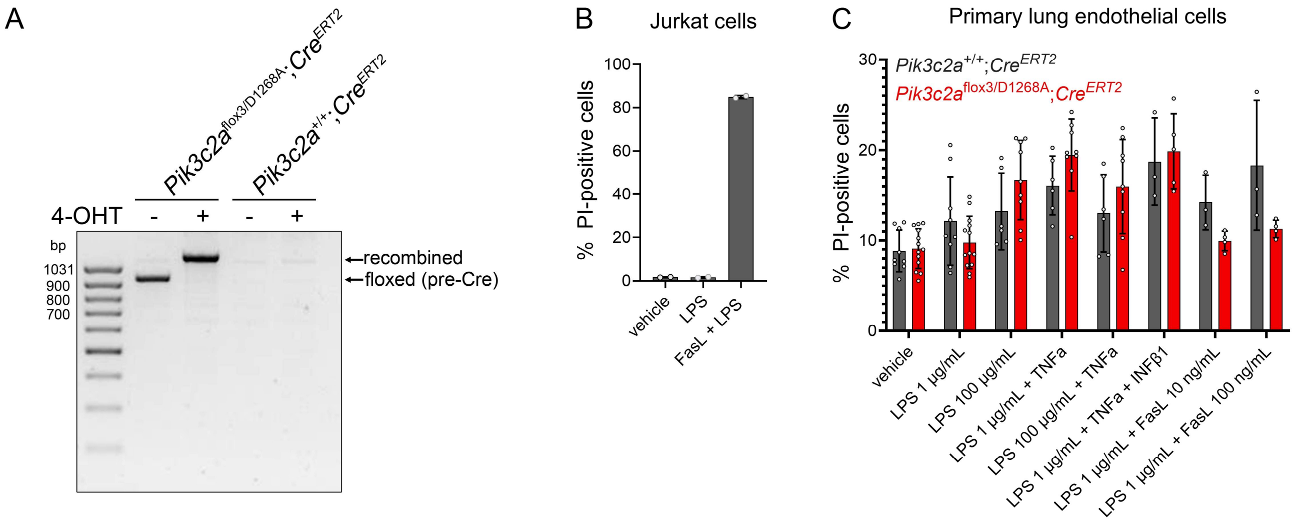

Fig. S5. Induced cell death in primary lung endothelial cells. (A) Analysis of Cre^{ERT2}-mediated recombination in primary lung endothelial cells treated with 4-OH-tamoxifen (4-OHT) for 48 h. Genomic DNA was isolated from primary lung endothelial cells from *Pik3c2a*$^{+/+}$;*Cre*^{ERT2} or *Pik3c2a*$^{flox3/D1268A}$;*Cre*^{ERT2} mice and recombination efficiency was assessed by PCR-amplification of a region surrounding the distal loxP site. The expected size of the PCR product before Cre-mediated recombination is 957 bp and after recombination 1170 bp. Note that the primers used are specific to the flox3 allele and do not bind to wild-type genomic DNA. (B) To verify the biological activity of the preparation of recombinant isoleucine-zipper-tagged FasL, the FasL-sensitive Jurkat T-cell line was treated with 1 µg/mL LPS or 1 µg/mL LPS + 10 ng/mL FasL for 72 h. Cells were labelled with propidium iodide (PI) and the percentage of PI-positive dead cells was determined by flow cytometry. Bars show mean ± s.d. from two biological replicates. (C) Primary lung endothelial cells from *Pik3c2a*$^{+/+}$;*Cre*^{ERT2} or *Pik3c2a*$^{flox3/D1268A}$;*Cre*^{ERT2} mice were treated as indicated for 72 h (TNFα: 10 ng/mL; interferon β1: 100 ng/mL). Cells were labelled with PI and the percentage of PI-positive dead cells was determined by flow cytometry. Bars show mean ± s.d. from primary lung endothelial cell cultures obtained from n = 3-8 (*Pik3c2a*$^{+/+}$;*Cre*^{ERT2}) or n = 4-12 (*Pik3c2a*$^{flox24\text{-}25/D1268A}$;*Cre*^{ERT2}) mice as indicated.

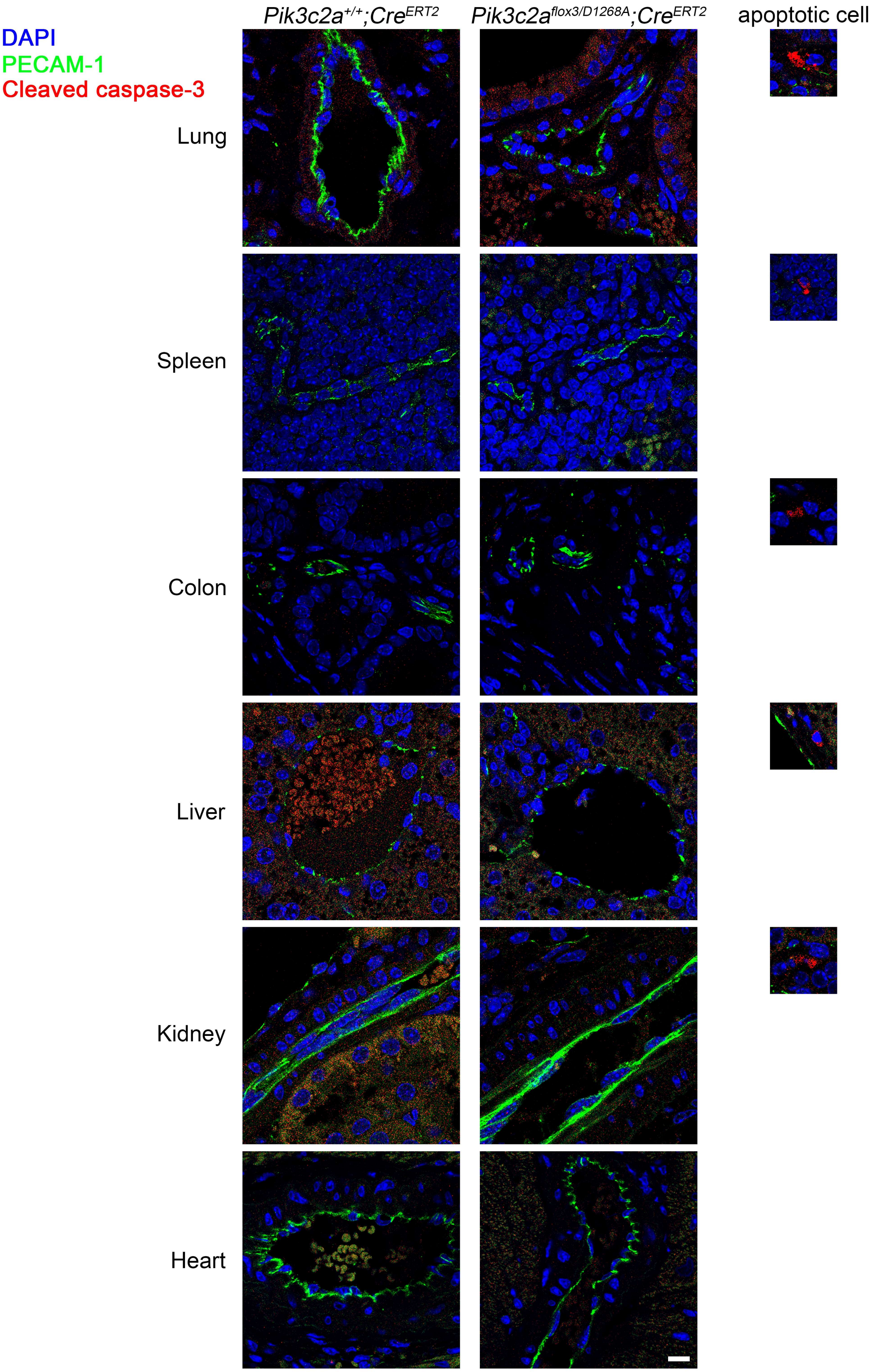

Pik3c2a+/+;CreERT2
Pik3c2aflox3/D1268A;CreERT2
apoptotic cell
DAPI
PECAM-1
Cleaved caspase-3
Lung
Spleen
Colon
Liver
Kidney
Heart

Fig. S6. Cleaved caspase-3 immunohistochemical staining of tissues after LPS challenge. *Pik3c2a*$^{wt/wt}$;*Cre*^{ERT2} (n = 3) and *Pik3c2a*$^{flox3/D1268A}$;*Cre*^{ERT2} (n = 4) mice were challenged with LPS. Mice were sacrificed 24 h post LPS injection when *Pik3c2a*$^{flox3/D1268A}$;*Cre*^{ERT2} mice became moribund. Tissues were stained for the vascular endothelial marker PECAM-1 and cleaved caspase-3 and counterstained with DAPI to label nuclei. Shown are representative images from one animal per tissue and genotype. The total number of PECAM-1-positive cells imaged across all fields of view per genotype were (*Pik3c2a*$^{+/+}$;*Cre*^{ERT2} / *Pik3c2a*$^{flox3/D1268A}$;*Cre*^{ERT2}): lung (1511 / 1494); spleen (860 / 1426); colon (615 / 1074); liver (505 / 761); kidney (1161 / 1823); heart (527 / 799). We did not observe PECAM-1 and cleaved caspase-3 double-positive cells in any condition. The cropped images on the right show arbitrary apoptotic cells observed in the same tissue in a separate field of view as a positive control for cleaved caspase-3 staining. Note that cells contained in the lumen of some vessels are non-nucleated red blood cells. Bar, 10 µm.

Table S1. Tissues included in histopathological analysis. Cohort included four female and four male mice each per genotype.

Adrenals
Aorta, Oesophagus
Brain
Brown fat pad
Caecum, Colon, Rectum
Duodenum, Pancreas, Stomach
Epididymides, Testes, Ovaries/oviducts
Eyes, Harderian glands, Optic nerve
Femur/Stifle joint
Gall Bladder, Liver
Heart
Ileum, Jejunum
Kidneys
Lung
Skin & Mammary glands
Mesenteric lymph nodes, Spleen, Thymus/Thymic area
Parathyroids, Thyroids
Prostate, Seminal Vesicles, Uterus/Vagina/Cervix, Urinary Bladder
Salivary gland, Submandibular Lymph Nodes, Tongue
Skeletal muscle
Spinal cord
Sternum
Perigenital fat pad
Perirenal fat pad

Table S2. Primary antibodies used

antigen	host species	used in Fig.	IHC dilution	blotting dilution	manufacturer	order number / reference
PECAM-1	rat	1B,C	1:250		BD Bioscience	553370
cleaved caspase-3	rabbit	1B,C	1:250 / 1:300	1:1,000	Cell Signaling Technology	9664 / clone 5A1E
PI3K-C2α	mouse	2B, S3D		1:200	BD Bioscience	611046
vinculin	mouse	2B, S3D		1:10,000	Merck (Sigma-Aldrich)	V9131
PI3K-C2α	mouse	2G		1:200	Santa Cruz Biotechnology	sc-365290
Hsp70	mouse			1:5,000	Invitrogen (ThermoFischer)	MA3-006
α-tubulin	mouse			1:4000	Sigma	T5168
PI3K C2β	mouse	S3D		1:250	BD Transduction	611342
phospho-IκBα (Ser32)	rabbit	S3D		1:1,000	Cell Signaling Technology	9241
phospho-NF-κB (S536)	rabbit	S3D		1:1,000	Cell Signaling Technology	3033
NF-κB total	rabbit	S3D		1:1,000	Cell Signaling Technology	3034
phospho-Erk1/2 (Thr202/Tyr204)	mouse	S3D		1:2,000	Cell Signaling Technology	9106
Erk1/2	rabbit	S3D		1:1,000	Cell Signaling Technology	9102
phospho-p38 (T180/Y182)	rabbit	S3D		1:1,000	Cell Signaling Technology	9215
p38 total	rabbit	S3D		1:1,000	Cell Signaling Technology	9212
phospho-JNK (Thr183/Tyr185)	mouse	S3D		1:1,000	Cell Signaling Technology	9255
Clathrin heavy chain	rabbit	S3D		1:1,000	Abcam	ab21679
PECAM-1	goat	S5	1:100		Santa Cruz Biotechnology	sc-1506

Table S3. Genotyping primers

allele	primer designation	sequence (5'-3')	amplified DNA
Pik3c2a^{D1268A}	C2a D1268A Flp fw	GACTGATTGGGATACAAACCC	WT 320 bp<br>D1268A 452 bp
	C2a D1268A Flp rev	GCTCTGAGCTGCAGATATGG	
Pik3c2a$^{flox24\text{-}25}$	Forward: oligo1 (6732_32)	CAGGATCTCATTCTAACTGGCC	WT 319 bp<br>flox pre Cre 372 bp<br>flox post Cre 409 bp
	Forward: oligo3 (6732_36)	TTTAGATTACAGCTATGAATCCCC	
	Reverse: oligo4 (6732_37)	AACGTAAAATCCCAATCTCCC	
Pik3c2a^{flox3}	C2a ex3 WT F	TAGACATCCTGGTTTATTAGAGTCC	WT 637 bp<br><br>C2aflox ex.3 preCre: 799 bp (WT primers) + 954 bp (flox primers)<br><br>C2aflox ex.3 postCre: 1170 bp (WT F + flox R)
	C2a ex3 WT R	CTTGTAGAAGAGAACTTCCAGTTG	
	C2a ex3 flox F	GCGCAACGCAATTAATGATAAC	
	C2a ex4 flox R	CCTGACAACTTGAGTGTGTTCC	
Cre^{ERT2}	Forward	GAATGTGCCTGGCTAGAGATC	190 bp (Cre-positive) and no product (Cre-negative)
	Reverse	GCAGATTCATCATGCGGA	
LysM-Cre	Wild-type specific: oIMR3068	TTA CAG TCG GCC AGG CTG AC	WT ~350 bp<br>Cre+ ~700 bp
	Common: oIMR3067	CTT GGG CTG CCA GAA TTT CTC	
	Transgene-specific: oIMR3066	CCC AGA AAT GCC AGA TTA CG	
Casp8$^{-/-}$	Caspase-8 fw	TTGAGAACAAGACCTGGGGACTG	WT 777 bp<br>KO 200 bp
	Caspase-8 rev	GGATGTCCAGGAAAAGATTTGTGTC	
Ripk3$^{-/-}$	RIP3 Newton common R	AGA AGA TGC AGC AGC CTC AGC T	WT 130 bp<br>KO ~480 bp
	RIP3 Newton WT F	ACG GAC CCA GGC TGACTT ATC TC	
	RIP3 Newton KO F	GGC ACG TGC ACA GGA AAT AGC	
Mlkl$^{-/-}$	MLKL fw	CAAGGAAAGAAGAACCTGCCCGA	WT 252 bp<br>KO 243 bp
	MLKL WT rev	GGATGCTGGCTGGCTGACA	
	MLKL KO rev	CCTGCTGCCATCTGAAACGG	
Tnfr1$^{-/-}$	TNFR-1 rev	GGCTGCAGTCCACGCACTGG	WT 472 bp<br>KO 300 bp
	TNFR-1 WT fw	TGT GAA AAG GGC ACC TTT ACG GC	
	TNFR-1 KO fw	ATTCGCCAATGACAAGACGCTGG	
Tie2-Cre	Tie2-Cre F	CGCATAACCAGTGAAACAGCATTGC	~500 bp (Cre-positive) and no product (Cre-negative)
	Tie2-Cre R	CCCTGTGCTCAGACAGAAATGAGA	

Table S4. Flow cytometry antibody panels

**Myeloid panel**					
**antigen**	**fluorophore**	**Supplier and order number (#)**	**concentration [mg/mL]**	**channel**	**dilution**
dead cells	APC-e780	eBioscience #65-0865-18	n.a.	R 780/60	1 µL/mL
CD45	V500	BD # 561487	0.2	V525/50	1:200
CD11b	BV421	Biolegend # 101251	0.2	V 450/50	1:200
Ly6G	APC	Biolegend #127613	0.2	R670/30	1:333
Ly6C	BV605	BD #563011	0.2	V610/20	1:200
B220	PE	Biolegend #103207	0.2	YG 586/15	1:100
CD3	FITC	Biolegend #100305	0.5	B530/30	1:200
**T-cell panel**					
dead cells	APC-e780	eBioscience #65-0865-18	n.a.	R 780/60	1 µL/mL
CD3	PE-Cy5	Biolegend #100310	0.2	YG 670/30	1:200
CD4	BV605	Biolegend #100547	0.05	V610/20	1:150
CD8	BV650	Biolegend #100742	0.2	V670/30	1:300
B220	PE	Biolegend #103207	0.2	YG 586/15	1:100
CD25	BV421	Biolegend #102043	0.2	V450/50	1:160
Ki67	PE-Cy7	Biolegend #652425	0.2	YG780/60	1:160
CD69	FITC	eBioscience #11-0691-81	0.5	B530/30	1:200
FoxP3	Alexa700	eBioscience #56-5773-82	0.2	R730/45	1:200
**Death receptor panel A**					
dead cells	APC-e780	eBioscience #65-0865-18	n.a.	R 780/60	1 µL/mL
CD31	Alexa488	BioLegend #102413	0.5	B 530/30	1:100
mTLR4	PE	Biolegend #145403	0.2	YG 586/15	1:100
CD95	PE-Cy7	BD #557653	0.2	YG 780/60	1:100
**Death receptor panel B**					
dead cells	APC-e780	eBioscience #65-0865-18	n.a.	R 780/60	1 µL/mL
CD31	Alexa488	BioLegend #102413	0.5	B 530/30	1:100
TRAIL-R2	PE	Biolegend #119906	0.2	YG 586/15	1:100
TNF-R1	APC	Biolegend #113006	0.2	R 670/30	1:100
**Death receptor panel C**					
dead cells	APC-e780	eBioscience #65-0865-18	n.a.	R 780/60	1 µL/mL
CD31	Alexa488	BioLegend #102413	0.5	B 530/30	1:100
IFNAR-1	PE	BioLegend #127311	0.2	YG 586/15	1:100

Extended methods

Isolation of primary cells

Primary mouse embryonic fibroblasts (MEFs) were obtained from E13.5 embryos from timed pregnancies with the day of the copulation plug counted as E0.5. Decapitated embryos were minced, and cells dissociated using trypsin. Single cell suspensions were allowed to adhere to culture dishes in DMEM with 4.5 g/L glucose, 10% fetal bovine serum and 100 U/mL penicillin/streptomycin at 37°C and 5% CO_2. Early passage primary MEFs (P2 to P5) were immortalized by retroviral transduction with a p53-shRNA construct and selected with 2 µg/mL puromycin for 10-14 days. For induction of Cre-mediated recombination, cells were treated with 1 µM 4-OH-tamoxifen (Merck Millipore, 508225) for 24 h.

For isolation of bone-marrow derived macrophages (BMMs) and dendritic cells (BMDCs), mice were euthanized and femur and tibia bones were dissected and cleaned. Using ice-cold culture medium (RPMI 1640, 1× penicillin/streptomycin, 2 mM L-glutamine, 50 µM β-mercaptoethanol, 10% fetal bovine serum) and a 26-gauge needle, the bone marrow was flushed out and collected in a 15 mL tube. Cells were dissociated by pipetting and passed over a 40 µm cell strainer. For differentiation into macrophages, 4-5 × 10^7 bone marrow precursor cells per non-tissue culture-treated 15 cm dish were kept in presence of 20% L929 cell conditioned medium (LCCM) for 8-10 days, refreshing the LCCM supplementation at least every three days. Cre-mediated recombination was induced by addition to 1 µM 4-OH-tamoxifen for the first 16-20 h in culture and fully removed thereafter. LCCM was prepared by seeding 300,000 L929 cells in 60 mL of complete DMEM (DMEM 4.5 g/L glucose, 1× penicillin/streptomycin, 2 mM L-glutamine, 10% fetal bovine serum) per T175 cm^2 flask and filtering (0.22 µm) the supernatant after 12 days in culture. LCCM aliquots were stored at -80°C.

For differentiation into BMDCs, bone marrow precursor cells were seeded to non-tissue culture treated dishes at 1× 10^6 cells/mL in presence of 200 ng/mL recombinant Flt3L (eBioscience, 34-8001-82) and 1 µM 4-OH-tamoxifen and cultured for 7 days before seeding cells for experiments.

Primary mouse lung endothelial cells were isolated as described in (1). Briefly, lungs were collected from 8-week-old mice, minced and disassociated using Dispase II (Roche Diagnostics, 4942078001). The Dispase II was neutralized with DMEM containing 10% FBS and 100 U/mL penicillin/streptomycin and cells washed twice with PBS containing 0.5% BSA (Labclinics, A0296). Endothelial cells were selected via incubation with CD144 antibodies (BD Pharmingen, 555289) and sheep α-Rat IgG Dynabeads (Life Technologies, 11-035) for 1 h at room temperature. Dynabead-bound endothelial cells were collected by magnetic separation and washed three times with PBS containing 0.5% BSA before seeding into gelatine-coated dishes in DMEM F-12 containing ECGS (Promocell, C-30120), 100 U/mL penicillin/streptomycin and 20% FBS. Once endothelial cells reached confluence a second CD144/α-Rat IgG Dynabead-mediated selection was performed to further enrich for endothelial cells. Cre-mediated recombination was induced by 2 µM 4-OH-tamoxifen treatment for 48 h, which was then removed and cells incubated for a further 5-8 days prior to seeding for experiments.

Immortalized cell culture

Jurkat cells were cultured in RPMI-1640 Medium (Thermo Fisher, 61870-036) with 10% FBS at 37 °C and 5% CO_2.

In vivo induction of Cre^{ERT2}-mediated recombination

Tamoxifen (Sigma-Aldrich, T5648) was dissolved fresh on each day of administration. The required amount of tamoxifen for a 15 mg/mL solution was weighed into a microcentrifuge tube, 10% of final volume ethanol was added and vortexed for about 1 min. Corn oil was added to the final volume and vortexed to give a clear solution with some undissolved crystals. This solution was incubated shaking at 37°C for about 15 min, manually inverting the tubes about every 3 min, until all crystals had fully dissolved. Mice at 7-10 weeks of age were administered 75 mg/kg tamoxifen per day by oral gavage using disposable feeding needles (Linton Instrumentation, FTP-20-38-50) for 2× 5 days, either for two weeks straight (weekdays only, weekend without treatment) or with one week of a break over the course of three weeks.

Immunohistochemical staining of yolk sacs

Yolk sacs from E10.5 embryos were dissected and fixed in 4% paraformaldehyde in phosphate buffered saline (PBS) overnight at 4°C without agitation. The next morning, yolk sacs were washed twice for 5 min in PBT (PBS + 0.5% Triton X-100) and blocked in PBT + 10% goat serum for 2 h at 4°C, using concave glass staining dishes. Yolk sacs were incubated with primary antibodies α-PECAM-1 at 1:250 (2 µg/mL) and α-cleaved-Caspase-3 at 1:250 in blocking solution overnight at 4°C (1 ml per dish; see supplementary table 2).

SDS-PAGE and Immunoblotting

Cultured cells were washed once in ice-cold PBS and scraped in lysis buffer (20 mM Hepes pH 7.4, 100 mM KCl, 2 mM $MgCl_2$, 1% Triton X-100, 1% protease and phosphatase inhibitor cocktails [Calbiochem]). After 5-15 min on ice, lysates were centrifuged at 20,800× g at 4°C for 3 min and cleared supernatants were collected.

Mouse tissues were harvested, snap-frozen in liquid nitrogen and stored at −80°C. Tissue were homogenized using Lysing Matrix M tubes (MP Biomedicals, Santa Ana, CA, USA) in lysis buffer on a FastPrep 24 homogenizer (MP Biomedicals) at 4 m/s for 20 s and immediately centrifuged at 20,800× g at 4°C for 5 min and cleared supernatants were collected.

Protein concentration in cleared lysates was determined by Bradford assay and samples were adjusted to 1× Laemmli sample buffer, heated to 95°C for 5 min and stored at -20°C. Samples were resolved by SDS-PAGE and transferred to nitrocellulose membranes using wet blotting (Bio-Rad) or dry blotting (iBlot2, ThermoFisher Scientific). Primary antibodies (see supplementary table 2) were diluted in 3% BSA, 0.02% NaN_3 in TBS and incubated on the blots shaking at 4°C overnight or for two days. Secondary HRP-conjugated antibodies were diluted in 3% milk powder in TBS and incubated on the blots for 1-2 h at room temperature. Chemiluminescence was detected with a LAS4000 digital imaging system (GE Healthcare).

In vitro kinase activity assay

Endogenous PI3K-C2α kinase activity was assayed in primary MEFs essentially as described (2). In brief, MEFs were harvested from 225 cm^2 flasks at 3 or 5 days after induction of recombination using 4-OH-tamoxifen. Endogenous PI3K-C2α was immunoprecipitated from cell extracts using home-made anti-PI3K-C2α serum SK193 (3) or rabbit-α-mouse IgG (Sigma-Aldrich M-7023) as a negative control. Kinase activity of immunoprecipitates was assayed in 50 µL reactions in kinase buffer (5 mM HEPES / KOH pH 7.2, 25 mM KCl, 2.5 mM magnesium acetate, 150 mM potassium glutamate, 10 µM $CaCl_2$, 0.2% CHAPS, 100 µM ATP) with 10 µg of L-α-phosphatidylinositol-4-phosphate (Avanti Polar Lipids, 840045) lipid substrate and 5 µCi γ-^{32}P-ATP (Hartmann Analytic, SRP 401) per reaction. The kinase reaction was allowed to proceed for 10 min at 37°C and lipids were extracted by methanol-chloroform extraction. Extracted lipids were separated by thin layer chromatography (stationary phase: Partisil K6, Silica gel 60 Å, Whatman [Schleicher & Scuell, 4860-820]; mobile phase: $CHCl_3$:Acetone:MeOH:acetic acid:H_2O at 64:30:24:30:13). γ-^{32}P signal on dried chromatography plates was detected using phosphor screens.

Immunohistochemical staining of formalin fixed paraffin embedded mouse tissue sections

Mouse lung, liver, kidney, spleen, colon, heart and gastrocnemius were formalin fixed, paraffin embedded and sectioned. Sections were dewaxed and rehydrated followed by antigen retrieval in 10 mM Sodium Citrate pH 6 sub-boiling in a microwave for 7 min, followed by permeabilization with 0.2% Triton X-100 diluted in Animal-Free Blocker and Diluent, R.T.U (Vector Laboratories, SP-5035-100) for 10 min and washed 2 x 5 min. Blocking was then performed using Animal-Free Blocker and Diluent, R.T.U for 1 hour at room temperature. Primary antibodies PECAM-1 (Santa Cruz, sc-1506) at 1:100 and Cleaved Caspase 3 (Cell Signalling, 9664) at 1:300 were diluted in Animal-Free Blocker and Diluent, R.T.U and incubated on sections overnight at 4 °C. Following 3 × 10 minute washes, fluorescently labelled secondary antibodies diluted in Animal-Free Blocker and Diluent, R.T.U were incubated with sections for 1 hour at room temperature, then sections were washed 6 × 10 min. Finally, sections were counter stained with DAPI (Roche, 10236276001) and mounted using ProLong Gold antifade reagent with DAPI (ThermoFisher Scientific, P36935). Sections were imaged by confocal microscopy using a Zeiss LSM 880 microscope with a ×63 oil Plan-Apochromator 1.4 numerical aperture objective lens, PMT detector, 405 nm, 458 nm, 488 nm,

514 nm, 561 nm, 594 nm and 633 nm lasers and Zen Black acquisition software with 512 x 512 pixels/image.

Immunophenotyping of mice by flow cytometry

Immunophenotyping was performed on cells harvested from blood, spleen and lymph nodes. Blood cells were collected as described above for clinical chemistry analysis of blood plasma, using the 400× g pellet and red blood cell lysis buffer (incubation on ice for 2 min; eBiosience, 00-4333-57) to remove erythrocytes. Spleen and lymph nodes were dissected and single cell suspensions generated by pressing the tissue through 70 µm cell strainers, followed by removal of erythrocytes using red blood cell lysis buffer for the spleen samples. 1× 10^6 cells per sample were stained for flow cytometry using a myeloid or T-cell antibody panel (see supplementary table 4 for a complete list of antibodies and dilutions) using BD Horizon Brilliant Stain buffer (BD, 563794) and rat-α-mouse FcγRII/III blocking antibody (eBioscience, anti-mouse CD16/32, 14-0161-82). For FoxP3 staining, the FOXP3 Fix/Perm Buffer Set from BioLegend (421403) was used.

Analysis of death receptor surface levels by flow cytometry

Primary mouse lung endothelial cells were gently detached using 2.9 mM EDTA over 1 h as described (4). Cells were stained for flow cytometry, using a fixable viability dye (eBioscience, 65-0865-18) to discriminate live and dead cells and rat-α-mouse FcγRII/III blocking antibody (eBioscience, anti-mouse CD16/32, 14-0161-82) to block immunoglobulin-binding surface receptors. Surface-exposed receptors were labelled using directly conjugated antibodies (see supplementary table 4 for details) and CD31- (PECAM-1) labelling was employed to confirm endothelial cells were being analyzed. Cells were kept in the cold throughout, and live cells were measured on a BD Symphony flow cytometer.

Cell death assay by flow cytometry

Primary mouse lung endothelial or Jurkat cells were seeded in 96-wells and treated with 1-100 µg/mL LPS, 10 ng/mL TNFα (PeproTech, 315-01A), 1 µg/mL CD14 (Abcam AB167706), 100 ng/mL IFN-β1 (BioLegend, 581302), 10 µM Bortezomib (Selleck Chemicals, S1013) or 1-500 ng/ml isoleucine-zipper-tagged human FasL for 72 h. Culture supernatants were collected, cells were trypsinized (for endothelial cells) and pooled with the supernatants. Cells were stained with 2 µg/mL propidium iodide (Sigma, P4864) and percentage of propidium iodide-positive cells within the singlet population was determined using a NovoCyte Advanteon flow cytometer.

References

1. P. Kobialka *et al.*, PI3K-C2beta limits mTORC1 signaling and angiogenic growth. *Sci. Signal.* **16**, eadg1913 (2023).
2. Y. Posor *et al.*, Spatiotemporal control of endocytosis by phosphatidylinositol-3,4-bisphosphate. *Nature* **499**, 233-237 (2013).
3. S. Alliouachene *et al.*, Inactivation of class II PI3K-C2alpha induces leptin resistance, age-dependent insulin resistance and obesity in male mice. *Diabetologia* **59**, 1503-1512 (2016).
4. M. Kaur, L. Esau, Two-step protocol for preparing adherent cells for high-throughput flow cytometry. *BioTechniques* **59**, 119-126 (2015).
